# Bioenergetic profile and redox tone modulate in vitro osteogenesis of human dental pulp stem cells: new perspectives for bone regeneration and repair

**DOI:** 10.1186/s13287-023-03447-9

**Published:** 2023-08-22

**Authors:** Francesca Agriesti, Francesca Landini, Mirko Tamma, Consiglia Pacelli, Carmela Mazzoccoli, Giovanni Calice, Vitalba Ruggieri, Giuseppe Capitanio, Giorgio Mori, Claudia Piccoli, Nazzareno Capitanio

**Affiliations:** 1 Department of Clinical and Experimental Medicine, University of Foggia, 71122 Foggia, Italy; 2 Laboratory of Pre-Clinical and Translational Research, IRCCS-CROB, Referral Cancer Center of Basilicata, 85028 Rionero in Vulture, Italy; 3 Clinical Pathology Unit, “Madonna delle Grazie’’ Hospital, Matera, Italy; 4 Department of Translational Biomedicine and Neuroscience “DiBraiN”, University of Bari “Aldo Moro”, 70124 Bari, Italy

**Keywords:** Human dental pulp stem cells, Osteogenesis/odontogenesis, Cell bioenergetics, Redox signaling, Antioxidants

## Abstract

**Background:**

Redox signaling and energy metabolism are known to be involved in controlling the balance between self-renewal and proliferation/differentiation of stem cells. In this study we investigated metabolic and redox changes occurring during in vitro human dental pulp stem cells (hDPSCs) osteoblastic (OB) differentiation and tested on them the impact of the reactive oxygen species (ROS) signaling.

**Methods:**

hDPSCs were isolated from dental pulp and subjected to alkaline phosphatase and alizarin red staining, q-RT-PCR, and western blotting analysis of differentiation markers to assess achievement of osteogenic/odontogenic differentiation. Moreover, a combination of metabolic flux analysis and confocal cyto-imaging was used to profile the metabolic phenotype and to evaluate the redox tone of hDPSCs.

**Results:**

In differentiating hDPSCs we observed the down-regulation of the mitochondrial respiratory chain complexes expression since the early phase of the process, confirmed by metabolic flux analysis, and a reduction of the basal intracellular peroxide level in its later phase. In addition, dampened glycolysis was observed, thereby indicating a lower energy-generating phenotype in differentiating hDPSCs. Treatment with the ROS scavenger Trolox, applied in the early-middle phases of the process, markedly delayed OB differentiation of hDPSCs assessed as ALP activity, Runx2 expression, mineralization capacity, expression of stemness and osteoblast marker genes (*Nanog, Lin28, Dspp, Ocn*) and activation of ERK1/2. In addition, the antioxidant partly prevented the inhibitory effect on cell metabolism observed following osteogenic induction.

**Conclusions:**

Altogether these results provided evidence that redox signaling, likely mediated by peroxide species, influenced the stepwise osteogenic expansion/differentiation of hDPSCs and contributed to shape its accompanying metabolic phenotype changes thus improving their efficiency in bone regeneration and repair.

**Supplementary Information:**

The online version contains supplementary material available at 10.1186/s13287-023-03447-9.

## Background

Adult stem cells are present in most of the organs in the human body, playing the indispensable role of ensuring maintenance and repair of the tissues in which they reside, such as bone marrow, peripheral blood, skin, skeletal muscle, adipose tissue and dental pulp [1, 2]. Among these tissues, dental pulp is considered as a rich and easily accessible source of mesenchymal stem cells (MSCs) suitable for tissue engineering applications and stem cell therapy [3, 4].

Human dental pulp stem cells (hDPSCs) exhibit self-renewal and multi-lineage differentiation abilities, including osteogenic, adipogenic, chondrogenic, myogenic and neurogenic differentiation [5, 6], remaining regenerative in adult teeth and responsive to various types of damage. Consequently, hDPSCs proliferate and migrate into the damaged area, differentiate in odontoblast-like cells and form reparative dentine [7–9].

The process of osteoblastic (OB) differentiation is regulated by many signal transducers in a complex signaling network. Among them, the extracellular signal-regulated kinase (ERK) pathway is of vital importance [10]. ERK is a member of the mitogen-activated protein kinase (MAPK) family, and its activation has been shown to promote osteogenic differentiation of osteoblast cells [11]. Accordingly, a majority of the in vitro inducers that modulate osteoblast activity appear to act in part through the ERK pathway [12]. Upon activation, ERK signaling triggers a series of downstream events that ultimately lead to the expression of osteogenic markers, such as osteocalcin (Ocn) and alkaline phosphatase (ALP). In addition, late OB markers, runt-related transcription factor 2 (Runx2) and osterix, are strongly regulated through ERK phosphorylation [13]. Consistently, mice with deletions of ERK1 and ERK2 display dramatically reduced bone mineralization [14].

Because of their highly proliferative, multi-differentiation potential and low immunogenicity, hDPSCs are considered as a promising source of stem cells for regenerative medicine and tissues engineering [9]. However, one fundamental issue regarding stem cells for regenerative medicine is maintenance of the stemness phenotype. Accordingly, the low presence of naïve stem cells in dental pulp isolates and the loss of functional and stemness properties during propagation limit their application in tissue engineering. Therefore, seeking a way to maintain the stemness of hDPSCs in cultures optimizing their properties would be of great significance [15].

An appropriate balance between self-renewal and differentiation is crucial for stem cell homeostasis and lineage commitment. A yet growing body of evidence highlights that this balance is partly regulated by reactive oxygen species (ROS)-mediated signaling in synchrony with aerobic metabolism [16–18]. The absolute cellular ROS content, their relative changes and compartmentalization, dictate cell fate by regulating various redox sensors thereby acting as physiological secondary messengers integrating environmental cues and cell-autonomous signaling [17, 19]. The presence of ROS balance within the stem cells is not only important for differentiation but also to keep their potency. Lower ROS content generally marks quiescence or self-renewal, with a more oxidized state marking cells with higher proliferation rates and lineage commitment [17, 20–22]. Under physiological conditions, stem cells maintain low levels of ROS to preserve their stemness and to remain quiescent in mammals. This notion has a practical outcome since reducing excessive ROS production proved to be a strategy for preserving stemness properties during long-term in vitro expansion of stem cells [23, 24].

Trolox (6-Hydroxy-2,5,7,8-tetramethylchromane-2-carboxylic acid), a water‐soluble derivative of vitamin E, is a well‐known phenolic antioxidant, scavenger of peroxyl and alkoxyl radicals. Trolox has been shown to protect against oxidative stress-induced damage in various cell types and animal models [25] exhibiting anti-cancer, cytoprotective, anti‐apoptotic, anti‐inflammatory, and vaso‐regulatory effects, which are all mediated through its ROS scavenging activity [26–29]. Previous studies have demonstrated that treatment with the antioxidant can affect stem cell properties retaining their differentiation potential [24]. In mice, Trolox has been shown to suppress osteoclast formation by downregulating receptor activator of NF-κB ligand (RANKL) induction and c-Fos expression [30]. All these findings suggest for Trolox a potentially relevant therapeutic utilization in regenerative medicine and stem cell-based therapies.

On the basis of what above-reported, the present study aimed to investigate the metabolic and redox profile of OB differentiating hDPSCs, with a focus on mitochondrial functions and to test the impact of Trolox on the balance between stemness maintenance and differentiative potential in an in vitro model of osteogenic induction.

## Methods

### Cell lines and culture conditions

Human dental pulp stem cells (hDPSCs) were isolated from wisdom teeth, in young adult healthy volunteers undergoing orthodontic treatments and characterized as previously described [31, 32]. The cells were cultured in α-Minimum essential medium (α-MEM) (Gibco, Gaithersbug, MD, USA) containing 5% fetal bovine serum (FBS) (Gibco), 1% antibiotics (100 U/mL penicillin-G, 100 µg/mL streptomycin) and 25 μg/ml amphotericin B; Gibco) in a humidified CO_2_ incubator at 37 °C as already reported [33]. The choice of a relatively low FBS content in the α-MEM was made to preserve the undifferentiated state under expansion culturing conditions (see also [33]). Primary hDPSCs were used between passages 3–6 and plated at a density of 5 × 10^3^ cells/cm^2^. For the antioxidant treatment, the cells were cultured in complete medium supplemented with 500 µM Trolox (6-Hydroxy-2,5,7,8-tetramethylchromane-2-carboxylic acid) (Sigma–Aldrich, St. Louis, MO). The Trolox-enriched medium was changed every 3 days. Untreated cells, used as control, were supplemented with vehicle only (DMSO) that never exceeded 0,002% (V/V). The cellular morphology was observed at an inverted optical microscope (Axio Vert A1, Zeiss, Oberkochen, Germany).

### Osteoblastic differentiation

For the induction of osteoblastic differentiation, the cells were cultured in mineralized-induced α-MEM medium containing 2% FBS, 50 µg/ml L-ascorbic acid, 10 mM sodium β-glycerol phosphate and 10 nM dexamethasone (Sigma–Aldrich, St. Louis, MO) for 7, 14 and 21 days as described in [33]. Fresh medium was replaced every three days and hDPSCs harvested for subsequent analysis at different time-points after induction of differentiation (0–7–14–21 days).

### Alkaline phosphatase (ALP) and Alizarin red S (ARS) staining

The ALP staining assay was performed using the Leucocyte Alkaline Phosphatase kit (Sigma–Aldrich, St. Louis, MO), based on napththol AS-BI and fast red violet LB, according to the manufacture’s protocol. Briefly, hDPSCs were cultured on a 12-well plate for differentiation in osteoblasts in mineralization-inducing medium in the presence or absence of Trolox for 7, 14 and 21 days. Then, medium was changed with 500 μl/well of the fixative solution and incubated for 30 s. After washing with sterile distilled water, 500 μl of ALP substrate solution were added to each well and left to react for 15 min at room temperature in the dark to stain the cells. ALP positive cells appeared stained purple. ARS staining was performed on day 21 to determine the osteogenic mineralization. The cells were fixed with 4% paraformaldehyde and then treated with 1% Alizarin Red S (pH, 4.1–4.3, Sigma–Aldrich, St. Louis, MO) for 30 min at room temperature protected from light. Then, the plate wells were rinsed twice with deionized water and air dried. For both ALP and ARS assays, all staining images were photographed, scanned and the intensity signal was evaluated using the ImageJ software (Wayne Rasband, NIH, USA, http://imagej.nih.gov/ij).

### Metabolic fluxes analysis

For real-time monitoring of the oxygen consumption rate (OCR) and extra-cellular acidification rate (ECAR) at different stages of osteogenic differentiation of hDPSCs, we used the XF96 extracellular flux analyzer (Seahorse Bioscience, Billerica, MA, USA) as previously described [34]. Briefly, cells were plated onto Seahorse 96-well plates 7, 14, 21 days before the experiment at a density of 5 × 10^2^ cells/well. Immediately before the experiment, cell culture medium was replaced with medium containing 25 mM glucose, 1 mM pyruvate and 2 mM glutamine, without serum. A baseline measurement of OCR and ECAR was taken, and then an inhibitory stress analysis was performed by consecutive injections of oligomycin (1 μM), carbonyl cyanide-p-trifluoromethoxyphenylhydrazone, FCCP (1 μM), rotenone + antimycin A (1 μM + 1 μM) and 2-deoxyglucose (100 mM). As detailed in the Results section and in the figure legend, the OCRs and ECARs assessed throughout the stepwise assay enabled to evaluate several bioenergetic parameters. The OCR and ECAR values were normalized to the protein content in each well, quantified by BCA assay (Thermo Scientific, Waltham, MA, USA).

### Live cell imaging of mtΔΨ and ROS

Cells cultured at different stage of differentiation (0–7–14–21 days) on fibronectin-coated 35-mm glass-bottom dishes (Eppendorf, Hamburg, Germany) were incubated for 20 min at 37 °C with either 2 μM TMRE (Tetramethylrhodamine ethyl ester perchlorate) to monitor mitochondrial membrane potential (mtΔΨ) or with 10 μM DCF-DA (2,7-dichlorofluorescin diacetate) or 5 μM MitoSox to evaluate intracellular peroxide and mitochondrial O_2_^•−^ respectively. In particular, MitoSox is a probe chemically engineered to target the mitochondrial matrix by adding a liposoluble cation (TPP^+^) moiety to hydroethidine which, once oxidized, enhances fluorescence following intercalation in double stranded DNA. Conversely DCF, derived from DCF-DA de-acetylation by cellular esterases, is a redox-sensitive probe, trapped within the cell in the cytosol as well as into mitochondria. Fluorescent dyes were purchased from Molecular Probes (Eugene, OR, USA). Stained cells were washed with PBS and examined by a Leica TCS SP8 confocal laser scanning microscopy system as described elsewhere [35] (images collected using a 60X objective [1.4 NA]). Acquisition, storage, and data analysis were performed using the Leica Application Suite integrated software (LAS-X, Leica Microsystems, Wetzlar, Germany) and further analyzed by the ImageJ software (Wayne Rasband, NIH, USA, http://imagej.nih.gov/ij).

### RNA extraction and real time PCR analysis

Total cellular RNA was isolated from hDPSCs at different stages of differentiation using TRIzol reagent (Invitrogen, Carlsbad, CA) according to the manufacturer’s instructions. RNA samples were treated with DNaseI (Invitrogen, Carlsbad, CA) and quantified using Nanodrop ND-1000 (Thermo Scientific, Waltham, MA, USA). Reverse quantitative real‐time polymerase chain reaction (PCR) assays were performed as described elsewhere [36] using the SYBR Green mix on a Roche Light Cycler 480 real‐time PCR Instrument (Roche Diagnostics GmbH, Mannheim, Germany). The relative expression of the target genes was quantified using the comparative threshold cycle (2^−ΔΔCt^) method and normalized by *Gapdh* levels. Features of primers used for real-time PCR are listed in Table 1.

**Table 1:** Features of the primers used for real‐time polymerase chain reaction

Gene	Organism	*T*_ann_ (°C)	Length (bp)	NCBI ref seq or sequence
*Nanog*	Homo sapiens	55	90	NM_024865
*Myc*	Homo sapiens	60	129	NM_002467
*Lin28*	Homo sapiens	55	126	NM_024674
*Sox2*	Homo sapiens	60	64	NM_003106
*Alp*	Homo sapiens	55	137	Forward: GACCTCCTCGGAAGACACTC Reverse: TGAAGGGCTTCTTGTCTGTG NM_000478.4
*Dspp*	Homo sapiens	55	136	Forward: ATATTGAGGGCTGGAATGGGGA Reverse: TTTGTGGCTCCAGCATTGTCA NM_014208.3
*Gapdh*	Homo sapiens	60	135	Forward: AGGCTGAGAACGGGAAGC Reverse: CCATGGTGGTGAAGACG

### Western blot analysis

Aliquots of cell lysates, containing 40 μg of proteins, were subjected to SDS polyacrylamide gel electrophoresis and proteins were blotted on a polyvinylidene difluoride (PVDF) membrane using a Trans Blot Turbo Transfer System (Bio-Rad Laboratories, Hercules, CA, USA). Membranes were probed with the following primary antibodies Runx2, phospo-ERK1/2, ERK1/2 (1:2000, Cell Signaling Technology, Danvers, MA, USA), MitoProfile Total OXPHOS Human WB Antibody cocktail (1:500; Abcam Cambridge, UK) and β-Actin (1:5000; Sigma Aldrich, St. Louis, MO, USA). After incubation with a correspondingly suited horseradish peroxidase-conjugated secondary antibody (1:5000, Cell Signaling Technology, Danvers, MA, USA), chemiluminescent signals were developed using the Clarity Western ECL Substrate (Bio-Rad Laboratories, Hercules, CA, USA), acquired with the ChemiDoc imaging system XRS + (Bio-Rad Laboratories, Hercules, CA, USA) and analyzed for densitometry with the ImageJ Lab 4.1 software.

### Statistical analysis

Data are reported as the mean ± standard error mean (SEM) of at least three independent experiments. Data were compared by an unpaired Student’s-t-test or, when necessary, by 2-way ANOVA followed by a post-hoc Bonferroni test. Differences were considered statistically significant if *p*-values < 0.05. All analyses were performed using GraphPad Prism Software Version 5 (GraphPad Software Inc., San Diego, CA, USA).

## Results

### Characterization of hDPSCs osteogenic differentiation potential

Human dental pulp stem cells (hDPSCs) were isolated from wisdom teeth, cultured under condition preserving their undifferentiated state and immunophenotyped by flow cytometry for the presence of stem cell markers as previously described [32].

**Fig. 1:**
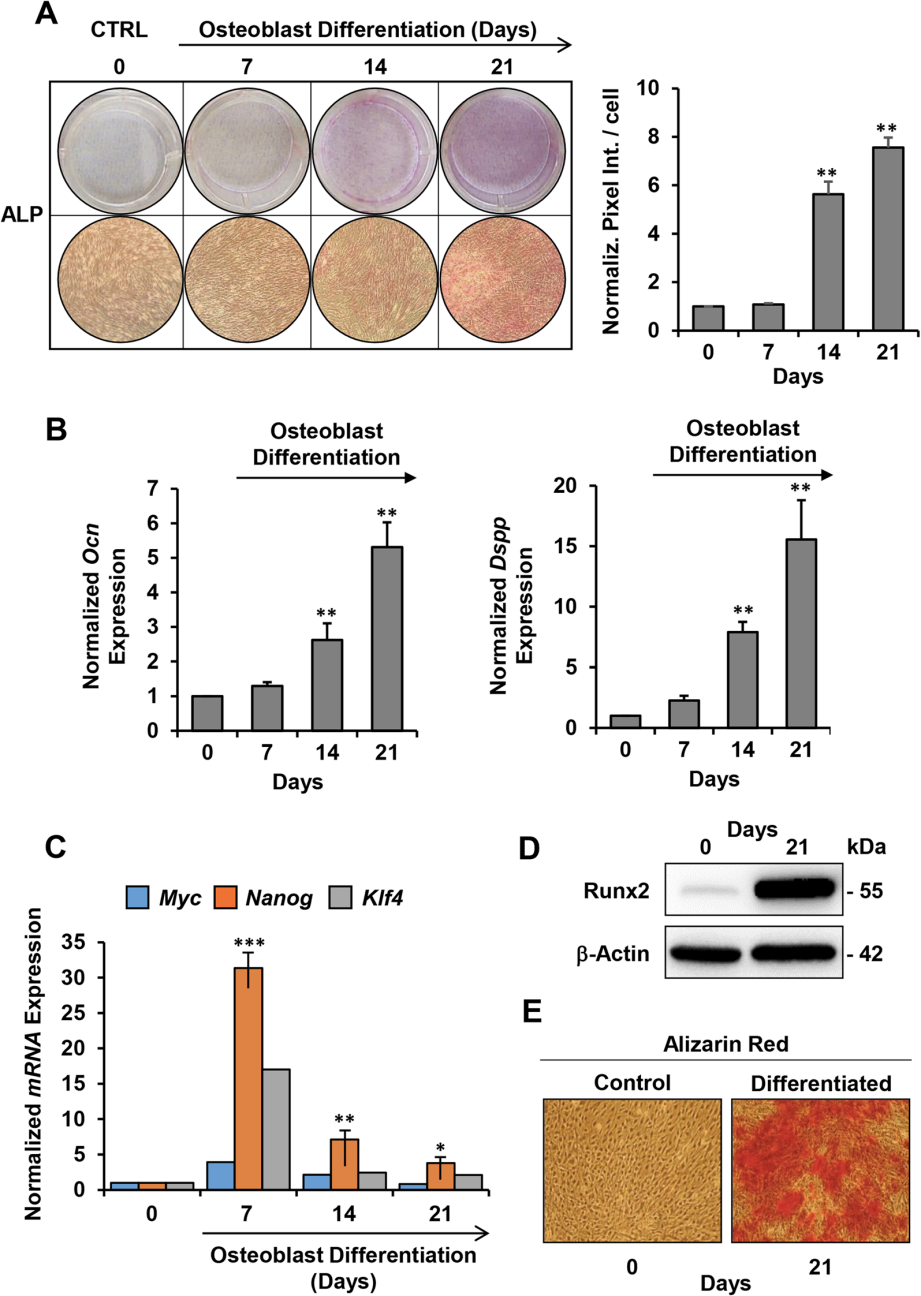
Osteogenic/odontogenic differentiation potential of hDPSCs. hDPSCs were induced to differentiate in osteoblast as detailed in Materials and Methods. Differentiating cells were stained or harvested at various time points (7, 14, 21 days) and undifferentiated cells (0 day) were taken as control. **A** Representative images of ALP staining during differentiation. The pictures on the left show photographs of ALP staining cells at the indicated time-points with relative enlarged microscopic inspection (at the bottom). The graph on the right displays densitometry analysis (Image J software) of ALP staining along with statistical evaluation; bars are means ± SEM of 4 biological replicates under each condition **P < 0.01 versus undifferentiated cells. **B** q-RT-PCR analysis of osteogenic specific gene markers *Ocn* and *Dspp* during OB differentiation of hDPSCs, normalized to their expression in undifferentiated cells; means ± SEM of 4–5 independent measurements (biological replicates) each carried out in 3 technical replicates; **P < 0.01 with respect to 0 day. **C** q-RT-PCR analysis of pluripotency gene markers *Myc, Nanog, Klf4* during OB-induction of hDPSCs; the bars are averages of three independent time-course experiments for *Nanog*; *P < 0.05; **P < 0.01; ***P < 0.005 versus undifferentiated. For *Myc* and *Klf4* the bars are averages of two independent measurements yielding comparable results. **D** Representative immunoblot of Runx2 at the indicated time-points. Full-length blots are presented in Additional file 1: Fig. S1. **E** Representative photographs of cells stained for extracellular calcification with Alizarin red at the indicated time-points

The differentiation potential of these adherent hDPSCs was examined in osteogenic culture conditions at 7, 14 and 21 days following supplementation of β-glycerol-phosphate, L-ascorbic acid and dexamethasone, a well-established differentiation procedure leading up to the mineralization step [33]. As shown in Fig. 1A, a time-dependent increase in alkaline phosphatase (ALP) activity was detected after the induction of osteogenic differentiation, as revealed by microscopy imaging of the ALP-stained cells. The quantitative analysis confirmed that the ALP activity increased by five- and sevenfold at 14 and 21 days of osteogenic induction. Consistently we further found that gene expression of osteocalcin (*Ocn*), a late osteogenic marker, as well as of the odontoblast differentiation marker dentin sialo phosphoprotein (*Dspp*) significantly and progressively increased along time points following OB differentiation, as shown by q-RT-PCR analysis (Fig. 1B). Intriguingly, expression analysis of well-known pluripotency-associated genes (*Myc, Nanog, Klf4*) resulted in significant increase of their transcripts, as compared with undifferentiated hDPSCs, in the earlier step of OB differentiation to decline suddenly in the later steps (Fig. 1C).

Finally, we observed that Runt-related transcription factor 2 (Runx2), the essential transcription factor for OB differentiation was overexpressed after 21 days of differentiation (Fig. 1D). Moreover, staining of the hDPSCs with Alizarin Red further confirmed their advanced differentiation along the osteogenic lineage, as demonstrated by large deposits of calcium phosphate (indicated by red-colored deposits of calcium phosphate) (Fig. 1E). Collectively, these data confirm the high osteogenic/odontogenic differentiation potential of hDPSCs in vitro.

### Analysis of mitochondrial features and energy metabolism during OB differentiation of hDPSCs

**Fig. 2:**
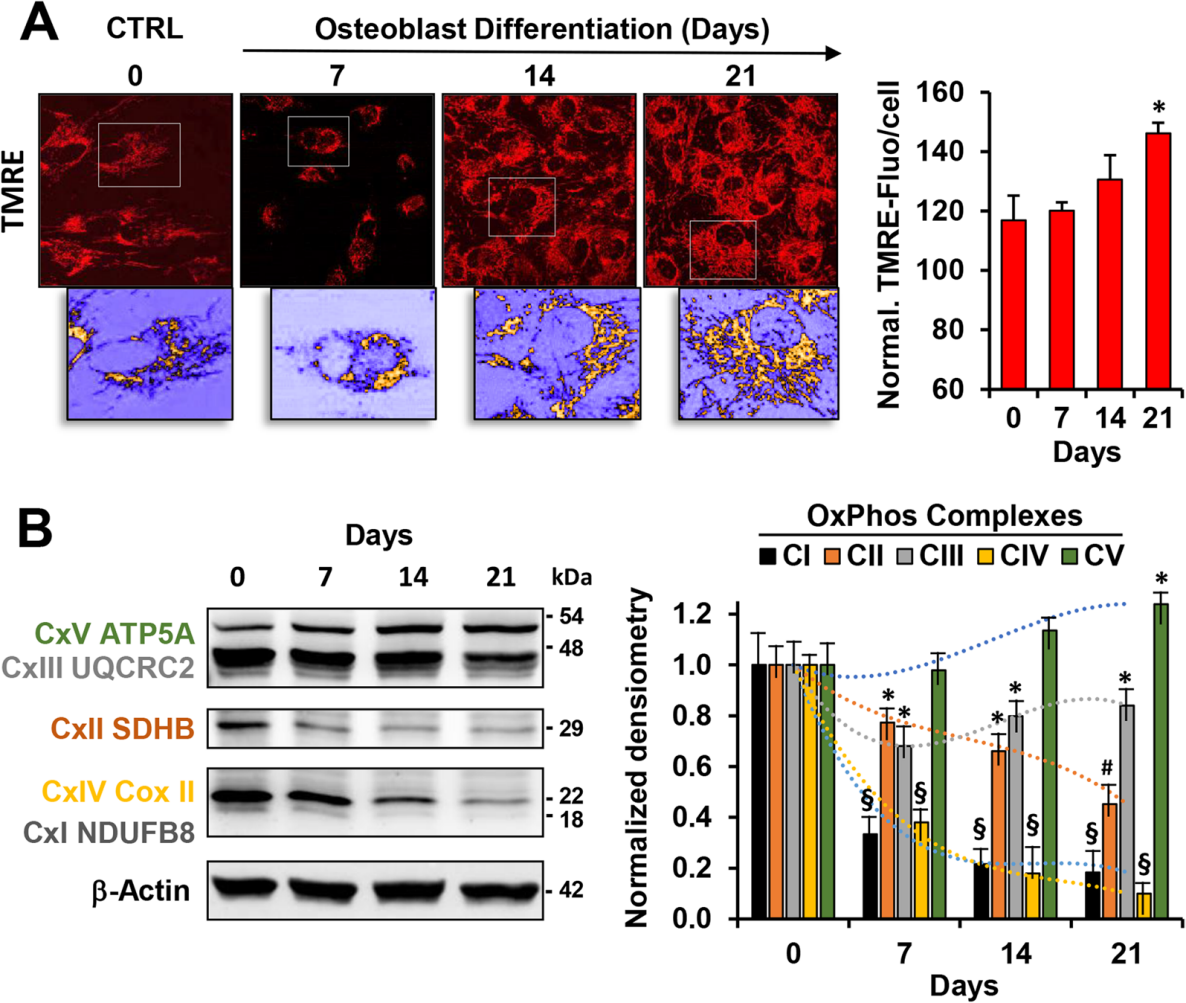
Mitochondrial morpho-functional analysis and evaluation of OxPhos complexes expression during OB differentiation of hDPSCs. **A** Representative laser scanning confocal microscopy imaging (LSCM) of mtΔΨ by the fluorescent probe TMRE in hDPSCs during OB differentiation (right panels); digital magnification of details is shown at the bottom of the pictures after false colour rendering and thresholding to remove background (ImageJ software). The histogram on the right shows the quantitative analysis of the TMRE-pixel density/cell; values are means ± SEM of three independent experiments under each condition where the digitalized fluorescence images from at least five randomly selected optical fields (each containing about 10–20 cells) were analysed by ImageJ tools; *P < 0.05. **B** Representative cropped immunoblotting of OxPhos complexes protein expression during OB differentiation and normalized densitometric analysis (right histogram) performed for three independent blots; Full-length blots are presented in Additional file 1: Fig. S2. β-actin was used as loading control; *P < 0.05; #*P* < 0.01; §*P* < 0.001 versus undifferentiated cells

Mitochondrial functions and energy metabolism are crucial for regulation of mesenchymal stem cells homeostatic properties such as stemness maintenance, proliferation and differentiation [37, 38]. Figure 2A shows a representative confocal microscopy analysis of the mitochondrial compartment attained by TMRE, a viable mitochondrial membrane potential (mtΔΨ) fluorescent probe, of hDPSCs at different time-points during osteogenic differentiation. TMRE accumulates electrophoretically in respiring mitochondria and therefore it is a probe pinpointing functionally active organelle. Staining revealed that, in the undifferentiated cells, the mtΔΨ-related TMRE signal displayed a diffused particulate appearance mainly spread in the cytoplasm, indicative of a prevalent fragmented rather than interconnected structure. A dissimilar feature resulted in the differentiated cells (at days 14 and 21) which displayed an annular peri-nuclear compartmentalization with the TMRE fluorescence localized in dense and highly interconnected mitochondria. Moreover, along with its morphological appearance, the OB differentiation caused a slight increase also in the intensity of the TMRE fluorescent signal at the latest stage of differentiation (Fig. 2A).

Next, we analyzed by western blot the expression levels of components of the mitochondrial oxidative phosphorylation system (OxPhos)—i.e. complexes I, II, III, IV and V (CI-CV)—using a cocktail of antibodies recognizing a specific subunit for each complex. Surprisingly, the protein expression levels of all the complexes of the respiratory chain resulted, counterintuitively with the TMRE imaging analysis, in a time-dependent decreased expression during OB differentiation. The changes were highly statistically significant for CI, CII and CIV, with those of CI and CIV occurring at day 7 post-differentiation start. Conversely, the expression of CV (FoF1-ATP synthase) resulted unchanged or slightly upregulated at later stages of OB differentiation (Fig. 2B).

**Fig. 3:**
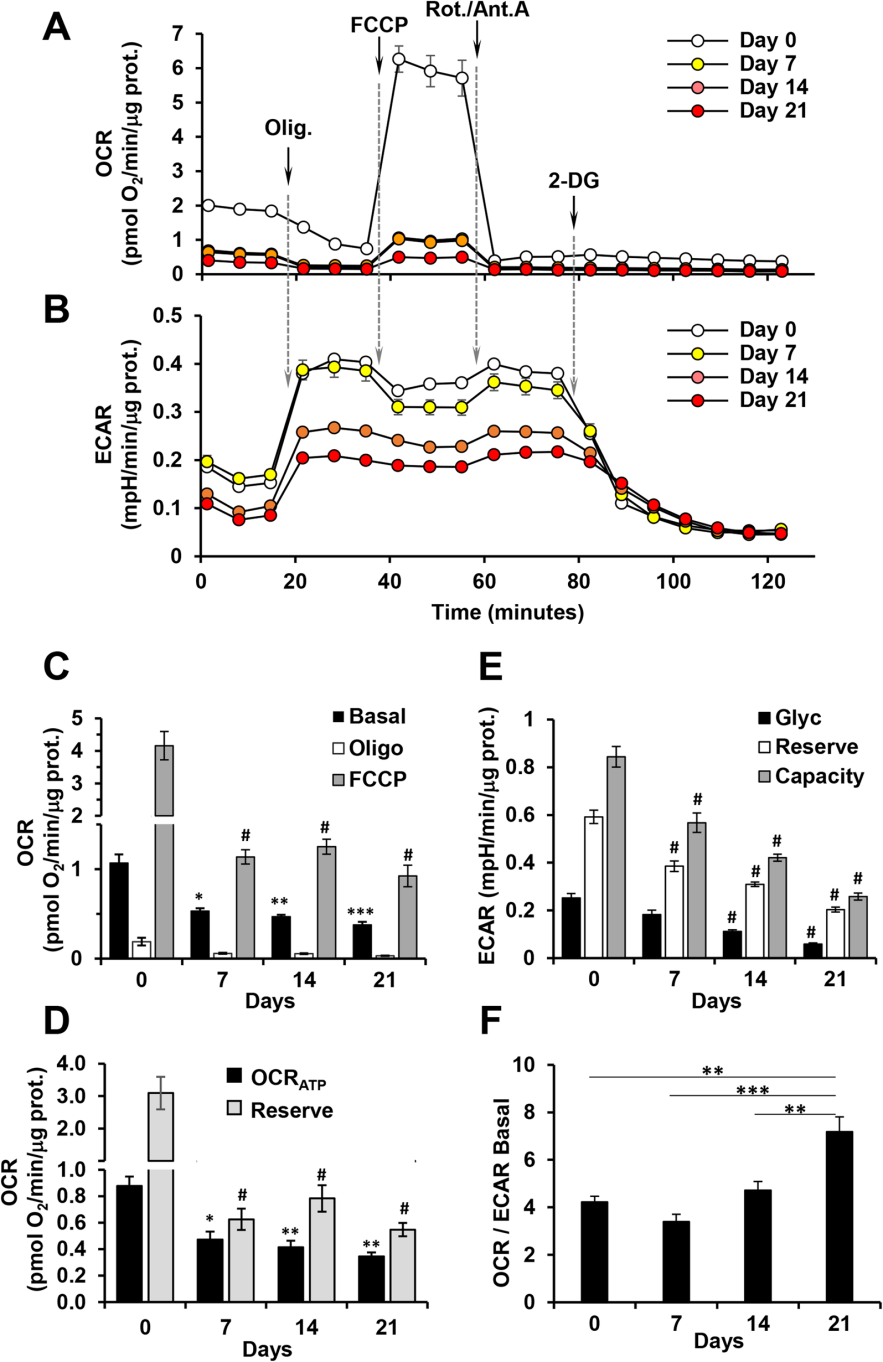
Metabolic flux analysis of hDPSCs during osteogenic differentiation. Representative oxygen consumption rates (OCR) (**A**) and extra cellular acidification rates (ECAR) (**B**) profiles of OB differentiating hDPSCs, assayed by Seahorse XFe96 Analyzer, at the indicated time-points as detailed in Methods section. Oligomycin (Oligo), FCCP, Rotenone/Antimycin A (Rot/Ant.A) and 2-deoxyglucose (2-DG) were sequentially added at the indicated arrows. The histograms in **C**, **E**, show OCR and ECAR respectively normalized to the protein content. **C** Basal, resting OCR; Oligo, OCR measured after the addition of the ATP synthase inhibitor oligomycin; FCCP, OCR measured after the addition of the uncoupler FCCP eliciting the maximal respiratory capacity. The shown OCRs were corrected for the residual OCR measured after the addition of the CI inhibitor rotenone. **D** Histogram showing OCR linked to ATP turnover (OCR_ATP_ = OCR_Basal_ − OCR_Oligo_)) and OCR Reserve (OCR_Reserve_ = OCR_FCCP_ − OCR_Basal_) as inferred from the OCR values reported in (C) as described in Methods section. **E** Glyc (Glycolysis), resting ECAR; Reserve, difference between ECAR measured in the presence of oligomycin and under resting conditions; Capacity, ECAR measured after the addition of oligomycin and refers to the maximal glycolytic activity with the OxPhos inhibited. The shown ECARs were corrected for the 2-DG-insensitive ECAR. **F** OCR/ECAR ratio under basal condition. All the bar values shown in (**C**–**F**) are means ± SEM of four independent experiments (biological replicates) carried out in 3 technical replicates under each conditions; *P < 0.05; **P < 0.005; ***P < 0.0005; #*P* < 0.0001 (versus undifferentiated condition for (**C**), (**E**), (**F**))

To deepen if and how cell metabolism was involved in the osteogenic differentiation of hDPSCs we analyzed the major metabolic fluxes by the Seahorse technology. Figure 3A, B show a representative time course assay whereby the oxygen consumption rate (OCR) and the extracellular acidification rate (ECAR) were assessed simultaneously on the same hDPSCs sample at different days of OB differentiation and under basal and different “stress” test conditions, i.e. after consecutive additions of the FoF1 ATP-synthase inhibitor oligomycin, the protonophore uncoupler FCCP, the CI/CIII combined inhibitors rotenone and antimycin A and the glycolysis inhibitor 2-deoxy-glucose (2-DG). As expected: (i) addition of oligomycin inhibited the basal OCR setting it at values depending on the H^+^-leak and caused a compensatory increase of ECAR (i.e. glycolytic flux); (ii) addition of FCCP largely collapsed the OCR-clamping mtΔΨ thus enhancing the respiratory rate to its maximal value and elicited a small inhibition of ECAR; (iii) addition of rotenone + antimycin A blocking the electron transfer enabled to appreciate the residual mitochondria-unrelated OCR and recovered ECAR to its maximal flux; (iv) the final addition of 2-DG blocked glycolysis allowing to appreciate the residual non glycolytic ECAR.

Figure 3C shows statistical analysis of the OCRs during osteogenic differentiation of hDPSCs. It can be clearly appreciated that the basal OCR is strongly dampened during differentiation starting even in its earliest phase (i.e. at day 7). Likewise, the maximal OCR activity elicited under uncoupling condition is strongly inhibited; the maximal OCR is an indirect parameter of the respiratory chain content in agreement with the results shown in Fig. 2B. Calculations on the parameters shown in Fig. 3C enabled to assess the OCR linked to the synthesis of ATP and the potential reserve activity confirming for both the occurrence of a strong down-regulation following the initiation of the osteoblastogenesis (Fig. 3D).

Statistical evaluation of the ECARs resulted in a much lower non-significant inhibition under basal conditions at day 7 post-differentiation start, that progressively increased during OB differentiation reaching statistical significance; conversely, the glycolytic reserve and capacity declined progressively from day 7 to day 21 (Fig. 3E). Combining the outcomes of the metabolic fluxes analysis, resulted that the OCR/ECAR basal fluxes ratio tended to increase during OB differentiation (Fig. 3F) although their absolute activities were substantially down-regulated (see also Fig. 6D ahead).

All together these results indicated the occurrence of relevant changes in the metabolic phenotype of hDPSCs undergoing osteogenic differentiation with a different contribution of the mitochondrial OxPhos and glycolysis in the earliest stage of the process.

### Evaluation of ROS during OB differentiation of hDPSCs

**Fig. 4:**
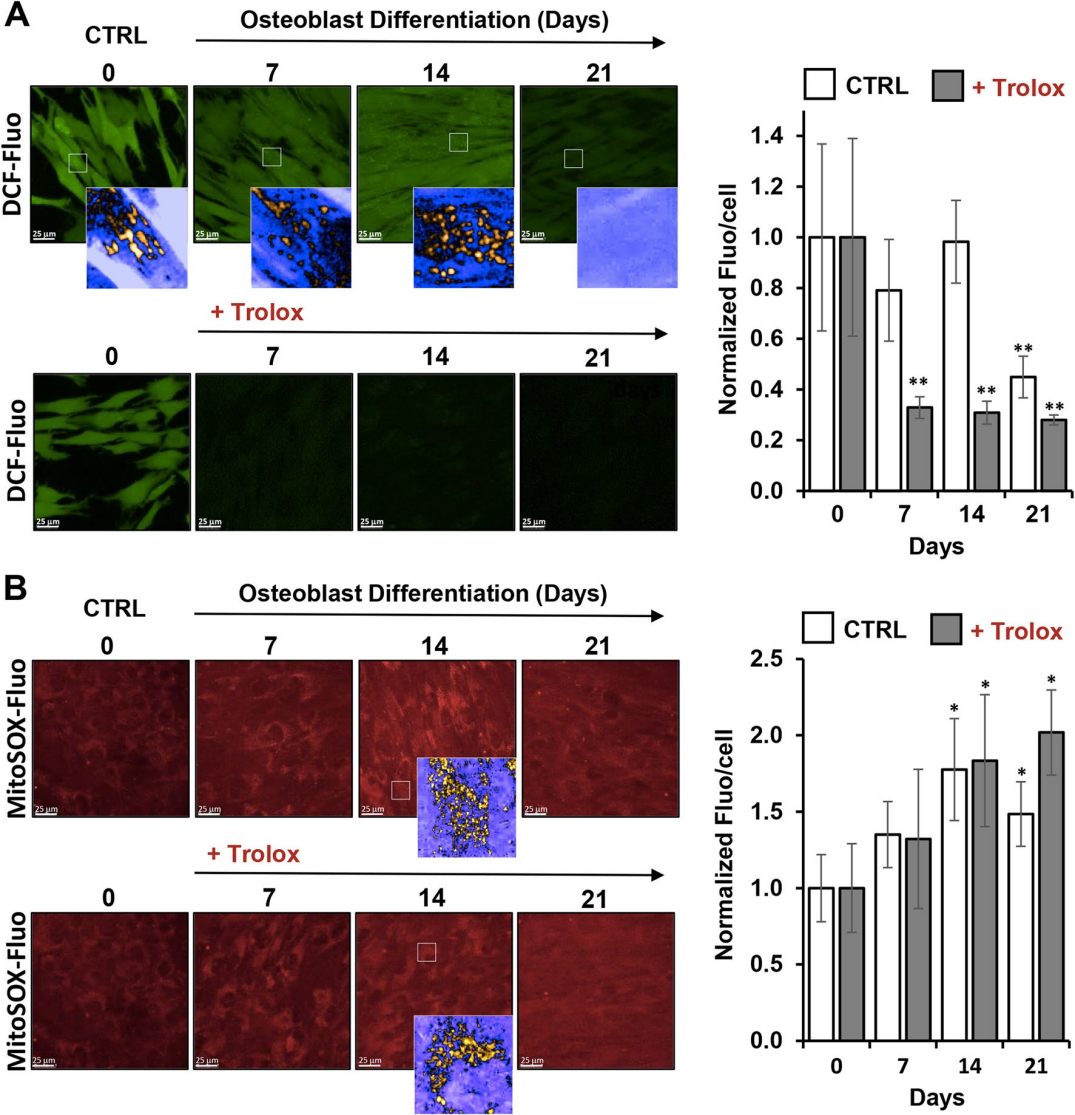
Evaluation of ROS content in hDPSCs undergoing OB differentiation. Representative LSCM imaging of ROS production during OB differentiation of hDPSCs assessed by DCF-DA (**A**) and MitoSox (**B**) probes, respectively. Where displayed enlarged details of the optical field (white square) rendered in false colours are shown at the bottom of the respective panel. The upper and lower sequences of images in (**A**) and (**B**) refer to differentiation in the absence and in the presence of the antioxidant Trolox (500 µM, present since the start of the OB induction), respectively. The images are representative of three different preparations yielding similar results. The histograms on the right of panels **A** and **B** show quantification of the fluorescence/cell in control and Trolox-treated cells, as elaborated by ImageJ; the bar values are means ± SEM of at least ten different optical fields, each containing 30–50 cells, from 3 independent experiments; *P < 0.05; **P < 0.001; versus untreated undifferentiated samples

As respiring mitochondria are a major source of cellular reactive oxygen species (ROS) and that changes in the mitochondrial activity are linked to alterations in ROS production [39, 40] we decided to measure ROS content in hDPSCs undergoing OB differentiation. To this aim we used the fluorescent probes dichlorofluorescein-diacetate (DCF-DA), a widely utilized probe to assess intracellular reactive species with a relative specificity toward peroxides, and MitoSox a mitotropic probe detecting mitochondrial superoxide anion (O_2_^•−^) production as further detailed in Methods section. Figure 4A, B show representative confocal microscopy imaging of hDPSCs at different days post osteogenic induction documenting that the basal DCF-related fluorescence signal remained unchanged up to the day 14 of differentiation as compared with the undifferentiated hDPSCs to drop significantly at day 21 post-induction.

A finer inspection of the images unveiled that the DCF-related fluorescence in addition to an intracellular spread appearance exhibited a brighter spotted signal nicely compatible with the mitochondrial compartment. Addition of 500 µM of the antioxidant Trolox, a water-soluble analogue of vitamin E, together with the osteogenic inducers, prevented almost completely the appearance of the DCF-fluorescence since the earlier steps of the differentiation process. Conversely, the MitoSox-related fluorescence (Fig. 4B) tended to increase progressively during the osteogenic differentiation reaching a significant difference at days 14–21 and no appreciable antioxidant activity was apparently exerted by Trolox. The different effect of Trolox might be explained in part with its relatively higher scavenging activity for peroxides as compared with superoxide [41] and in part with its limited propensity to cross the mitochondrial inner membrane.

### Effect of Trolox treatment on the OB differentiation potential of hDPSCs

Redox signaling is recognized to regulate the balance between self-renewal and proliferation/differentiation of stem cells [42, 43]. Therefore, we decided to investigate the role of ROS in OB differentiation of hDPSCs culturing them under osteogenic induction, but continuously treated with 500 µM Trolox.

**Fig. 5:**
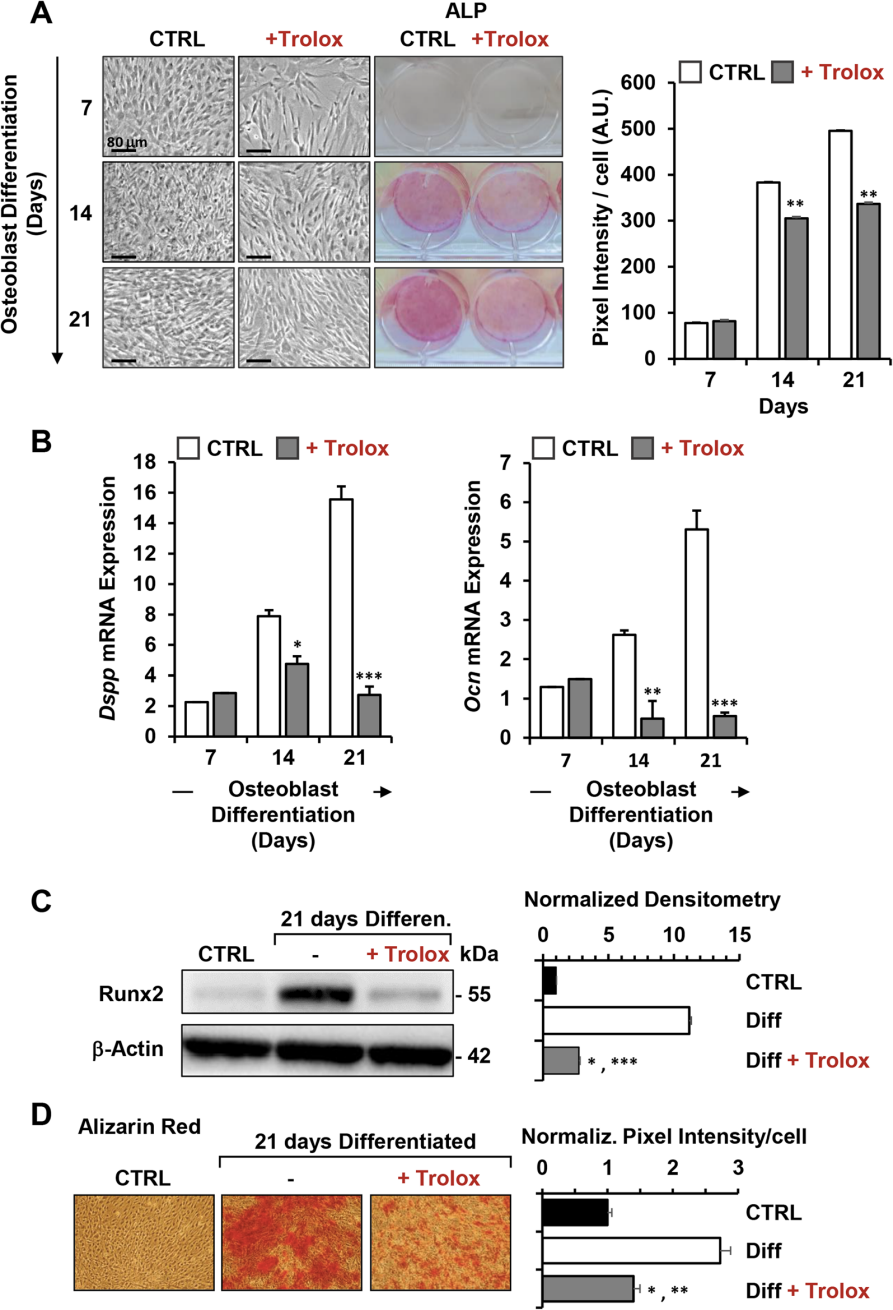
Effect of Trolox on the OB differentiation potential of hDPSCs. **A** Representative images of differentiating hDPSCs ± 500 μM Trolox (administered since the induction start) at days 7, 14 and 21 shown as phase-contrast (left half part of the panel) and photographs of ALP staining (right half part of the panel). Scale bar, 80 μm. The graph on the right displays normalized densitometry analysis (Image J software) of ALP staining along with statistical evaluation; bars are means ± SEM of 4 biological replicates under each condition **P < 0.01 versus untreated cells. **B** q-RT-PCR analysis of the osteogenic markers *Dspp* and *Ocn of* DPSCs cultured under osteogenic conditions ± 500 μM Trolox. The values are means ± SEM of normalized transcript levels of four independent biological experiments, *P < 0.05; **P < 0.01; ***P < 0.001 versus untreated cells. **C** Protein expression levels of Runx2, assayed by western blotting in undifferentiated (CTRL), untreated (-) and 500 μM Trolox-treated cells after 21 days from osteogenic induction. Left panel: a representative immunoblot. Full-length blots are presented in Additional file 1: Fig. S5. Right bar histogram: densitometry analysis normalized to β-actin as means ± SEM of three independent experiment; *P < 0.05 versus CTRL; ***P < 0.001 versus untreated differentiated cells. **D** Analysis of mineral matrix deposition assayed by Alizarin Red (red staining) in undifferentiated hDPSCs (CTRL), untreated (−) and 500 μM Trolox-treated cells after 21 days from osteogenic induction. The graph on the right shows quantitative analysis of alizarin red staining carried out by a densitometric analysis (Image J software); *P < 0.05 versus CTRL; **P < 0.01 versus untreated differentiated cells

Figure 5A shows the phase contrast imaging of Trolox-treated cells, displaying, along with a reduced cell growth, evidence of change in the osteoblastic-differentiated hDPSCs morphology towards a more pronounced stem cell like fibroblastoid morphology, particularly evident at day 7 and 14 of OB differentiation induction. Following Trolox treatment the ALP staining decreased significantly on day 14 and day 21 compared to the control, as shown by microscopy imaging and relative quantification of the ALP-stained cells. Accordingly, the expression of the odonto/osteogenic markers *Ocn* and *Dspp* was significantly reduced following Trolox treatment at days 14 and 21 post-induction (Fig. 5B). In addition, western blot analysis revealed that the antioxidant treatment fully prevented the enhanced expression of Runx2 (Fig. 5C). Finally, Trolox treatment caused a reduced mineralization capacity of differentiating hDPSCs at day 21 as shown by microscopy imaging of the alizarin red stained cells (Fig. 5D).

Altogether these findings clearly showed that the antioxidant Trolox dampened dramatically OB differentiation of hDPSCs in vitro.

### Effect of Trolox treatment on mitochondrial function during OB differentiation of hDPSCs

**Fig. 6:**
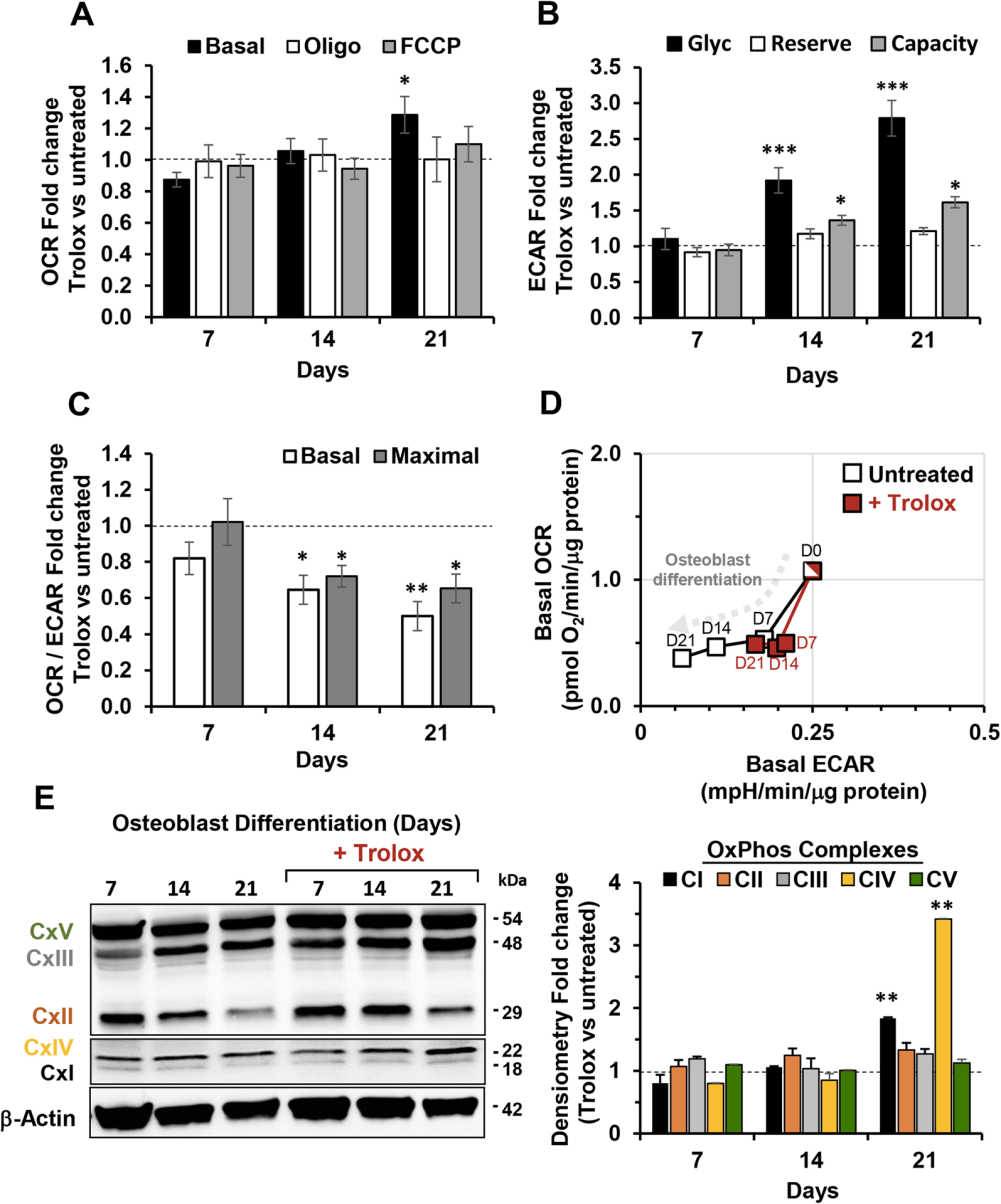
Effect of Trolox on mitochondrial function during OB differentiation of hDPSCs. hDPSCs cultured under osteogenic induction medium were continually treated with 500 µM Trolox and OCR (**A**) and ECAR (**B**) measured at 7, 14 and 21 days by Seahorse XFe96 Analyzer as in Fig. 3. **C** OCR/ECAR ratios under basal and maximal activities are shown. Bars values in (A)-(C) are means ± SEM of fold changes of Trolox-treated versus untreated of 3 biological replicates each carried out in triplicate; *P < 0.05; **P < 0.001; ***P < 0.0005. See Fig. 3 for abbreviations. **D** Energy map obtained plotting the basal OCR and ECAR shown in Fig. 3C, E for Trolox-untreated OB differentiating hDPSCs (white squares) and in panels A and B for Trolox-treated cells (red squares). The values shown are absolute activities and the features on each symbol indicate the differentiating time-points. **E** Representative cropped immunoblot of the OxPhos complexes protein expression in untreated and Trolox-tretaed cells using a cocktail of specific antibodies as detailed in Methods section. Full-length blots are presented in Additional file 1: Fig. S6. Histogram on the right shows the ß-actin-normalized densitometric analysis as fold changes of Trolox-treated versus untreated hDPSCs; bar values are means ± SEM of data resulting from three independent blots; **P < 0.001 versus untreated

Subsequently, we assessed the impact of Trolox treatment on the metabolic fluxes of hDPSCs during OB differentiation. Data revealed practically no changes of the OCRs, as compared with untreated hDPSCs, with the exception of a modest significant increase of basal OCR at day 21 of differentiated cells (Fig. 6A). Conversely, the treatment of hDPSCs with Trolox resulted in a progressive significant enhancement of ECAR at days 14 and 21 of both glycolysis and glycolytic capacity as compared with untreated cells, while the glycolytic reserve resulted not affected at the same time-points (Fig. 6B). These findings were highlighted in Fig. 6C that displays a time-dependent dampening of OCR/ECAR ratio under basal and maximal stimulated conditions in Trolox-treated cells, as compared with the untreated cells. Correlation of the basal OCRs and ECARs in the energy map shown in Fig. 6D clearly displayed that Trolox affected the metabolic profile in differentiating hDPSCs. Finally, OxPhos complexes (CI to CV) protein expression levels were significantly influenced by Trolox treatment only for the augmented expression of CI and CIV in differentiated cells at day 21, consistent with the increased OCR observed at the same time-point following antioxidant treatment (Fig. 6E).

Taken together these data suggested that the dampening of OB differentiation exerted by Trolox might be related to a metabolic rewiring in differentiating hDPSCs.

### Effect of Trolox on ERK pathway during OB differentiation of hDPSCs

**Fig. 7:**
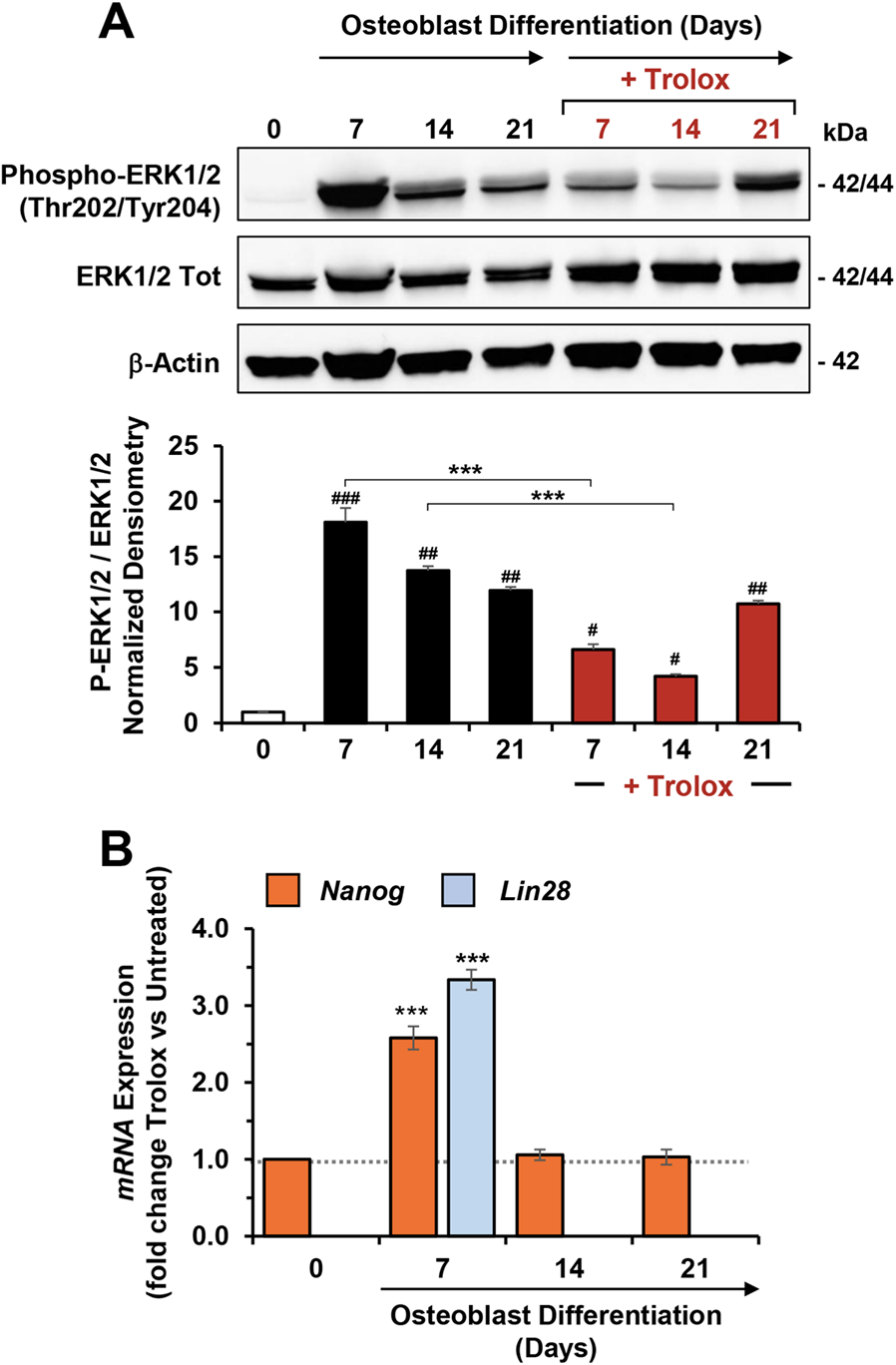
Effect of Trolox on ERK pathway and stemness-genes expression of hDPSCs during OB differentiation. **A** Representative immunoblotting for protein expression and phosphorylation of ERK 1/2 during OB differentiation of hDPSCs ± 500 μM Trolox (administered since the induction start) at 7, 14 and 21 days; full-length blots are presented in Additional file 1: Fig. S7. β-actin was used as loading control. Lower panel: densitometric analysis displaying the phospho-ERK1/2 (Thr202/Tyr204)/ERK1/2 ratio (normalized to β-actin), expressed as mean ± SEM of three independent experiments; #*P* < 0.05; ##*P* < 0.001; ###*P* < 0.0001 versus undifferentiated control (0 day); ***P < 0.001 Trolox-treated versus respective untreated. **B** q-RT-PCR evaluation of *Nanog* and *Lin28* gene expression following Trolox treatment in hDPSCs at the indicated time-points post OB induction; the bar values are fold changes of the relative expression of Trolox treated cells compared to untreated cells. Data are means ± SEM of GAPDH-normalized transcript levels of 3 independent biological experiment under each condition. **P < 0.01; ***P < 0.005

Activation of mitogen activated protein kinases (MAPKs) pathways proved to play an important role in differentiation of stem cells [44]. Therefore, we explored the involvement of extracellular signal-regulated kinases (ERKs) in the OB differentiation of hDPSCs and the impact on it of Trolox treatment. Figure 7A illustrates the result of immunoblot analysis carried out with antibodies recognizing the activated phosphorylated form (P-Thr202/P-Tyr204 ERK1/2) and the total amount of ERK1/2. It is clearly shown that, following induction of OB differentiation, ERK1/2 is strongly phosphorylated in the early step of the process (i.e. at day 7) to decline progressively in the later steps. Trolox treatment significantly reduced phosphorylation of ERK1/2 at days 7 and 14 with no effect at day 21.

Notably, Trolox treatment further enhanced the expression of the pluripotency-associated gene *Nanog*, at day 7 post OB induction, as compared with that observed in Trolox-untreated hDPSCs (Fig. 1C). At the same time-point of differentiation, the expression of another stemness gene marker, *Lin28*, resulted likewise significantly enhanced following Trolox treatment (Fig. 7B). No differences in the expression levels of both *Myc* and *Klf4* at each time-points post-induction in the Trolox-treated hDPSCs were apparently observed as compared with untreated cells (data not shown).

### Late exposure to the antioxidant Trolox delays OB differentiation potential of hDPSCs

**Fig. 8:**
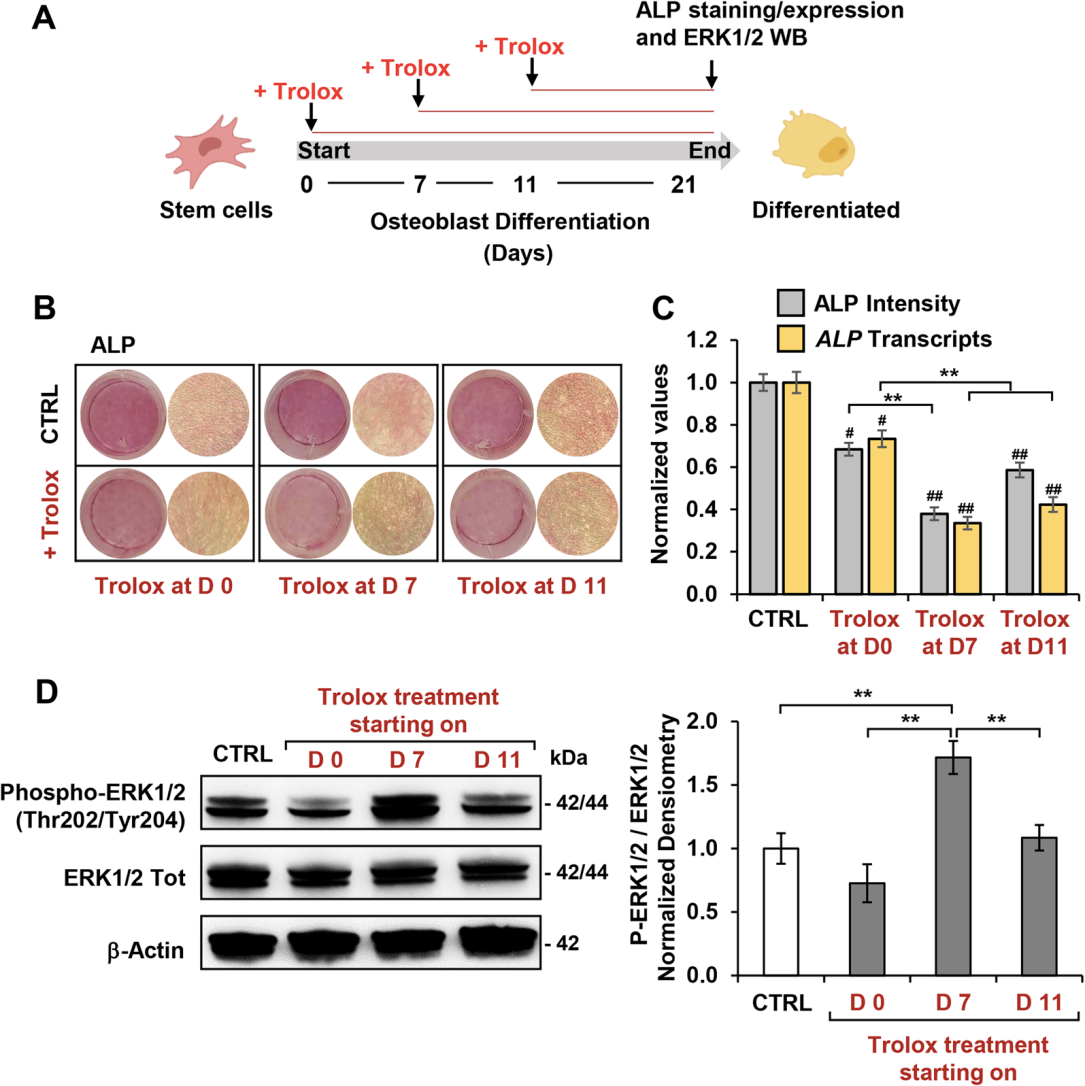
Effect of delayed Trolox treatment on OB differentiation potential of hDPSCs. **A** Schematic illustration of the experimental design. Trolox treatment of OB-differentiating hDPSCs started at days 0, 7 and 11 after administration of the inducers; the following analyses were carried out at day 21. **B** Representative photographs of ALP staining of untreated and Trolox-treated hDPSCs as illustrated in (**A**), and histograms (**C**) with densiometric analysis of ALP staining (grey bars) and *Alp* gene expression (yellow bars) performed for three independent experiments; #*P* < 0.05; ##*P* < 0.001; versus undifferentiated control; **P < 0.001 versus respective untreated. **D** Representative immunoblotting for protein expression and phosphorylation of ERK1/2 in untreated and Trolox-treated hDPSCs as illustrated in (**A**); full-length blots are presented in Additional file 1: Fig. S8. Right panel: densitometric analysis displaying phospho-ERK1/2 (Thr202/Tyr204) / Erk1/2 ratio (normalized to β-actin), expressed as means ± SEM of three independent experiments; **P < 0.001

The latest results suggested the occurrence in the OB differentiation process of different effective time-windows. In particular, the interval between days 7–14 post-induction appeared crucial in the multistep process anticipating the major phenotypical changes accompanying commitment. Therefore, to deepen this point we decided to investigate the impact of the antioxidant Trolox supplemented in a time-windows between the first and second week from the initiation of the hDPSCs OB differentiation (i.e. at days 0, 7 and 11 after administration of the inducers; Fig. 8A); the following analyses were carried out at day 21. Figure 8B shows that as compared with the reduction in the ALP activity caused by Trolox added at day 0, that observed with Trolox added at days 7 and 11 was even larger. Accordingly, *Alp* gene expression followed substantially the same trend of ALP activity resulting in a more pronounced reduction at days 7–11 of Trolox time-points as compared to Trolox time-point at day 0. No changes were observed of the metabolic fluxes (i.e. OCRs and ECARs) irrespective of the time-shift in the Trolox treatment (data not shown).

Finally, we assessed the phosphorylation state of ERK1/2 under the same out-of-phase protocol of Trolox treatment described above. Intriguingly, as compared with antioxidant-untreated OB differentiated hDPSCs, the phosphorylation of ERK1/2 was at day 21 significantly larger when Trolox was administered at day 7 post-induction.

Altogether these data would suggest the early phase of induction as a check-point-like step for the OB differentiation of hDPSCs.

## Discussion

Understanding the mechanism and conditions fostering stem cells to egress from their quiescent undifferentiated state is important not only to improve our basic knowledge in stemness biology but also to optimize the utilization of stem cells in regenerative medicine on which great hope is hold in the future. It is clearly emerging that commitment of both embryonic and adult stem cells is accompanied with profound metabolic changes to cope with the energy demand of lineage-specific differentiating cells. In this study we sought to investigate metabolic changes occurring following osteogenic induction in hDPSCs. Similar analyses have been carried out with mesenchymal stem cells (MSCs) isolated from different tissues such as bone marrow and adipose tissue [45, 46] but limited information is available in literature for hDPSCs.

Given the recognized role of mitochondrial activity in the cellular energetic metabolism we first assessed the mitochondrial content and cellular localization in hDPSCs undergoing osteoblastic differentiation attained with treatment of the well-characterized protocol consisting in treatment with dexamethasone/ascorbate/ β-glycerophosphate (D.A.G.). This treatment extended for 21 days enables hDPSCs to proliferate and to differentiate in osteoblasts finally undergoing hydroxyapatite mineralization (shown in Fig. 1). The functional mitochondrial content per cell appeared to slowly increase after day 7 of D.A.G. treatment reaching statistical significance at the end of the treatment. To notice the intracellular localization of mitochondria displayed an increasingly perinuclear appearance during OB differentiation in agreement with other reports [47] (Fig. 2A). This would be interpreted as a need for the differentiating cell to have rapidly available ATP for the nuclear activity from the mitochondrial compartment.

However, and surprisingly because counterintuitive with the previous finding, the protein expression of all the complexes of the oxidative phosphorylation system, with the exemption of the FoF1-ATP synthase (CV), decreased progressively during OB differentiation. The expression of complexes I (NADH dehydrogenase) and IV (cytochrome c oxidase) showed a more dramatic decline. This finding was confirmed by assessing the mitochondrial respiratory activity (i.e. OCRs) that decreased significantly even on day 7 post-induction and from here forward (Figs. 2B and 3A). The maximal OCR activity (elicited by FCCP), that is an indirect measure of the respiratory chain complexes content, decreased dramatically in good agreement with the respiratory chain complexes expression. However, in spite of the 75% reduction of the maximal OCR, that linked to ATP production was more contained (i.e. 50%). Conversely, the glycolytic flux, measured contemporary as ECAR on the same samples, although decreasing during OB differentiation displayed a different time-dependence with only a small if any effect in the first 7-day post-induction but a more dramatic inhibition in the terminal steps of differentiation (Fig. 3B).

All together these observations highlight the occurrence of a complex metabolic shift during OB differentiation with a rapid dampening of the mitochondrial respiratory competence in the first steps of the commitment followed by a much slower decline in the later steps. Conversely, glycolysis started to substantially decrease only in the later steps of OB differentiation. It is important to highlight that cultured MSCs, maintained in their undifferentiated state in appropriate medium (e.g. with low FBS content [31, 33]), following administration of commitment inducers undergo a proliferative stage that precedes the actuation of the differentiation program. Therefore, it is reasonable that the balance of the major energy-producing pathways does not follow identical trends during the whole differentiation process. Beyond the energy demand the production of intermediate metabolites as precursors of biosynthesized molecules might be different depending on the actual stage of the stem cells (i.e. expanding versus differentiating). In this sense, maintenance of the glycolytic flux might be important in generating precursors needed for the earlier proliferative phase whereas reduced expression of the respiratory chain complexes might be a strategy to divert upstream intermediate metabolites, such those of the tricarboxylic acid cycle, toward anaplerotic molecules needed in the differentiative stage. Indeed, a tight connection between metabolism and epigenetic modifications, such as those occurring during the differentiation program, is emerging [48–50].

Our findings nicely agree with what reported by others for hDPSCs [51], but conflict with what observed during OB differentiation of bone marrow-derived MSCs [52–54]. In bone marrow derived MSCs it was found that differentiated osteoblasts exhibited either enhanced or unchanged mitochondrial OCR and unchanged or reduced ECAR as compared with undifferentiated cells. Moreover, conflicting results among different studies are presented concerning the mtDNA copy number and the expression of nuclear- or mitochondria-encoded respiratory chain complexes proteins.

The reasons of the discrepancy in the measured metabolic fluxes might reside in the different protocol utilized. In our study hDPSCs were directly seeded in a multi-wells microplate and the start of OB differentiation was time-shifted so that all the samples at different stage of differentiation were Seahorse-assessed at the same time without transfer from culturing plates. Another possibility that cannot be ruled out is that MSCs isolated from different sources might adopt different strategies for the same lineage-specific commitment.

Aerobic metabolism is linked to the cellular redox balance. First by influencing the NAD(P)/NAD(P)H ratio and then by generating reactive oxygen species. These latter come from the diversion of the normal electron transfer throughout the mitochondrial chain directly to dioxygen with production of the one-electron reduced superoxide anion (O_2_^•−^) which is then dismutated to hydrogen peroxide (H_2_O_2_) [40]. Hydrogen peroxides differently from the charged superoxide anion is much more diffusible within the cell. In addition to the mitochondrial respiratory chain another important source of ROS is given by members of the NADPH oxidase (NOX) family located at the plasma membrane. NOXs deliberately reduce dioxygen to O_2_^•−^ which is then converted to H_2_O_2_ [17]. Initially believed as harmful by-products of the oxygen metabolism it is nowadays well accepted that below a given threshold (i.e. under physiological conditions or “oxidative eustress” [17]) ROS work as messenger-like in bio-signaling or can modulate cellular signaling pathways. The chemical basis of the major effect of ROS is their ability to oxidize reactive cysteines sulfhydryl groups in the proteins influencing their activities.

Regarding the stem cell biology, the reported role of basal ROS production varies between maintaining the self-renewal/potency of naïve stem cells to inducing differentiation toward a certain lineage. Moreover, the ROS level can vary depending on the differentiation stage. Beyond the different role played in various stem cell types, ROS are cell-proliferation inducers. Indeed, it has been shown that mitochondrial ROS drive cell cycle progression and proliferation by promoting the redox-mediated phosphorylation of CDK2 in the G1-S transition [55].

In keeping with these notions, we investigated the basal ROS content in OB differentiating hDPSCs and the impact on it of antioxidant treatment. The results attained show that the bona fide peroxide production in hDPSCs is maintained at the level of the undifferentiated cells in the first two weeks post-OB induction to decrease significantly in the late stage of differentiation (Fig. 4). This is consistent with the reported observation that antioxidant enzymes expression is up-regulated in the terminal step of the osteoblastic differentiation [52]. Although the probe we used did not enable us to identify the cellular source of ROS, nevertheless the brighter spotted signal within the cells would suggest the involvement of mitochondria in generating peroxide which then diffuse in the cytosol. However, other extramitochondrial ROS-sources cannot be excluded [17]. Conversely, utilization of a mito-tropic probe specific for the germinal ROS O_2_^•−^ would unveil an opposite trend with their level significantly increasing in the middle-later stages of OB differentiation. However, it should be highlighted that the fluorescent level of MitoSox might be influenced by the extent of the mitochondrial membrane potential (as shown by TMRE in Fig. 2A) and by changes in the mitochondrial DNA copy number likely occurring during hDPSCs expansion/differentiation. Notably, MitoSox fluorescence is enhanced following its intercalation in double stranded DNA [56, 57]. On this basis we would de-emphasize the apparently observed increase of MitoSox fluorescence given the above-mentioned biases.

Contrary to what in principle expected no clear correlation between ROS production (irrespective if H_2_O_2_ or O_2_^•−^) and the respiratory chain activity/content was evident.

In a previous study from our group, we reported that human mesenchymal stem cells isolated from different tissues, including hDPSCs, as well as hematopoietic stem/progenitor cells express detectable level of myoglobin utilizing non-muscle alternative transcripts [32]. In addition to the known function of myoglobin in the storage and delivery of oxygen, other functions have been reported such as controlling the homeostasis of reactive oxygen and nitrogen species [58] and vehiculation of fatty acids to mitochondria [59]. Notably, the protein expression of myoglobin resulted strongly down-regulated in the last week of the OB differentiation of hDPSCs [32]. This observation, along with what was reported in the present study, warrants further investigation in the context of the stem cell physiology.

Co-administration of the antioxidant Trolox with the osteogenic inducers (i.e. from day 0 to day 21) reduced significantly the cellular level of DCF-sensitive ROS since the earlier step of induction to the values observed in the latest stage but in untreated differentiating hDPSCs. Conversely, any effect of Trolox was observed on the MitoSox-sensitive ROS (Fig. 4). This result agrees with the notion of the relative higher selectivity of Trolox, a membrane permeant hydrophilic analogue of vitamin E, toward peroxides [41].

The results attained clearly showed that continuous exposure of hDPSCs to Trolox, since the beginning of the OB-inducing treatment, caused a significant inhibition of the osteogenic process. This was particularly evident in the middle-late stage of differentiation where, as compared with the antioxidant-untreated hDPSCs, the ALP activity and the expression of the osteoblast marker genes *Dspp* and *Ocn* were significantly inhibited as well as the expression of Runx2 and the mineralization capacity measured at the end of the treatment (Fig. 5). Notably, the antioxidant caused a significant up-regulation of the glycolytic flux or, better to say, prevented the inhibitory effect observed following OB induction. Again, this effect was evident in the middle-late stage of the process. Conversely, the osteogenesis-mediated inhibition of the mitochondrial respiratory activity was only partially prevented on the latest stage of the process as well as the expression of complexes I and IV (Fig. 6).

Altogether these results provided evidence that redox signaling, likely mediated by peroxide species, influenced the stepwise osteogenic differentiation of hDPSCs and contributed to shape its accompanying metabolic phenotype changes.

Mechanistic insights about the effect of Trolox were provided by monitoring the activation of ERK1/2. ERK1/2 are protein kinases belonging to the MAPK family whose activation depends on phosphorylation. In the context of osteogenesis, it has been shown that active P-ERK1/2 phosphorylate, thereby activating, the key osteogenic transcription factor Runx2 [60, 61]. Most notably several studies report redox-mediated phosphorylation of ERK1/2 either by activation of other kinases in the MAPK path or by inactivation of an ERK1/2-directed phosphatase [62]. Oxidation by peroxide of critical protein cysteines has been implicated in both cases.

In agreement with these notions, we found that under our experimental conditions, counterintuitively, ERK1/2 undergo strong phosphorylation in OB differentiating hDPSCs at day 7 post-induction to slowly decline in the later phases. Antioxidant treatment caused a significant inhibition in the phosphorylation of ERK1/2 more pronounced in the earlier and middle phases of the process (Fig. 7). This observed effect of Trolox might be consistent with a delayed time-shifted activation of ERK1/2 accounting for the observed inhibited actuation of the differentiative program. In other words, our interpretation is that an oxidative eustress provides a favorable condition for the action of differentiation-factors. Therefore, ROS might be envisaged, in this context, as sufficient but not necessary signaling molecules.

The presented results suggested the occurrence, in the first phases of OB-induction of redox-mediated events tuning the timing of a proper differentiative program. To test this hypothesis, we administered Trolox at days 7 and 11 after OB-induction. The result attained showed that at the later step of the “standard” commitment (i.e. at day 21) the osteogenic markers were far to be activated as compared with the antioxidant-untreated cells and even with those treated on day 0 with Trolox. Further, the phosphorylation state of ERK1/2 was at day 21 higher as compared either with the antioxidant-untreated cells and with those treated on day 0 (Fig. 8).

Altogether, the observations reported in this study highlight the occurrence on one side of a profound rewiring of the metabolic phenotype during differentiation of hDPSCs toward the osteo/odontogenic lineage and, on the other hand, a complex crosstalk between redox and osteogenic induction signaling in the same context.

Regarding the first point several studies report changes in the metabolic profile of stem cells undergoing differentiation [38, 53]. It is generally accepted that stem cells are characterized by a quiescent phenotype because of their limited energy demand. Moreover, glycolysis is the catabolic pathway preferred in place of the more efficient aerobic metabolism to limit ROS production that potentially might compromise the genomic integrity of the stem cell compartment. Such a metabolic choice is also conditioned by the relatively hypoxic tissutal environment where several stem cells reside. However, this notion cannot be generalized because of the extreme biological diversity of stem cells. Even without considering embryonic stem cells, within the adult MSCs a growing number of pluri/multi-potent stem cells is emerging residing in specialized niche in practically all the tissues [63, 64]. It is therefore conceivable that the basal phenotype in different MSC compartments might be different depending on their specific energy needs. Although osteodontogenesis of bone marrow-derived MSCs has been reported to increase progressively their mitochondrial aerobic metabolism, to the best of our knowledge only a study is reported with hDPSCs showing, instead, reduced OxPhos activity during OB differentiation [51]. Moreover, in that study no compensatory increase of the glycolytic flux was observed suggesting an apparent overall depression of the bioenergetic competence of differentiating hDPSCs. The results provided in the present study confirm and further extend those observations having been carried on a longer time-interval post OB-induction (i.e. for 21 instead of 14 days) suggesting a distinctive metabolic strategy of hDPSCs during osteogenesis. Moreover, transcriptomic analysis carried out by our group in the OB-differentiating hDPSCs has revealed that within the expected rearrangement of gene expression, enrichment analysis, based on GSEA method and MSigDB gene sets, identified a downregulated KEGG pathway pertaining to the mitochondrial oxidative phosphorylation. Notably, downregulation of OxPhos was observed as early as in the first week post OB-induction and increased only a little at the end of the differentiation process (normalized enrichment score (NES) of − 1.44, *P* < 0.009 and of − 1.66, *P* < 0.0003 at day 7 and day 21 versus undifferentiated, respectively) (manuscript in preparation).

The actuation of a differentiative program leading to specialized cell-types consists in a temporally sequence of steps with the earlier phase characterized, in principle, by self-renewal of the true stem cell and proliferation of the progenitor (one of the two stem cell daughters in case of an asymmetric division). Isolation of pluripotent cells from any tissue, even if phenotyped for the presence of specific stemness markers, never provides a highly homogeneous population of stem cells. Rather a mixture of naïve stem cells and un-committed progenitors are usually obtained and maintained in suitable culturing media. Substitution of the maintenance medium with one containing cocktails of lineage-specific factors boosts the differentiative process that last in vitro a few weeks. In the first phase post-induction stem cells/progenitors undergo cell division and proliferate. The expression profile shown in Fig. 1C would indicate that in this first phase proceeding the differentiative steps, cells need to maintain or even enhance their pluripotency phenotype. The entity and duration of this phase depends on the specific lineage-commitment. In the case of MSCs a comparative study showed that osteogenic differentiation induces a fast and robust proliferation that levels off after two weeks with the first week displaying the highest growth rate. Conversely, no or much more limited proliferation during neurogenesis or adipogenesis has been observed, respectively [65]. Only when the expansion phase declines the expression of specialization genes start to be operative. Consistently it has been reported that the time-coordination of cell proliferation and differentiation relies on the antagonism between cell cycle regulators and cell type-specific gene expression [66]. According to these notions we found that counterintuitively the expression of known stemness marker genes as well as phosphorylation of ERK1/2 are upregulated in the early expansion phase post-induction and start to decline only in the later differentiative phases (see also [31]. Most notably, dampening of OB differentiation by Trolox-treatment is accompanied by no changes or even up-regulation of stemness marker genes. In addition, and most notably, scavenging of ROS-mediated signaling delayed phosphorylation-mediated activation of ERK1/2.

**Fig. 9:**
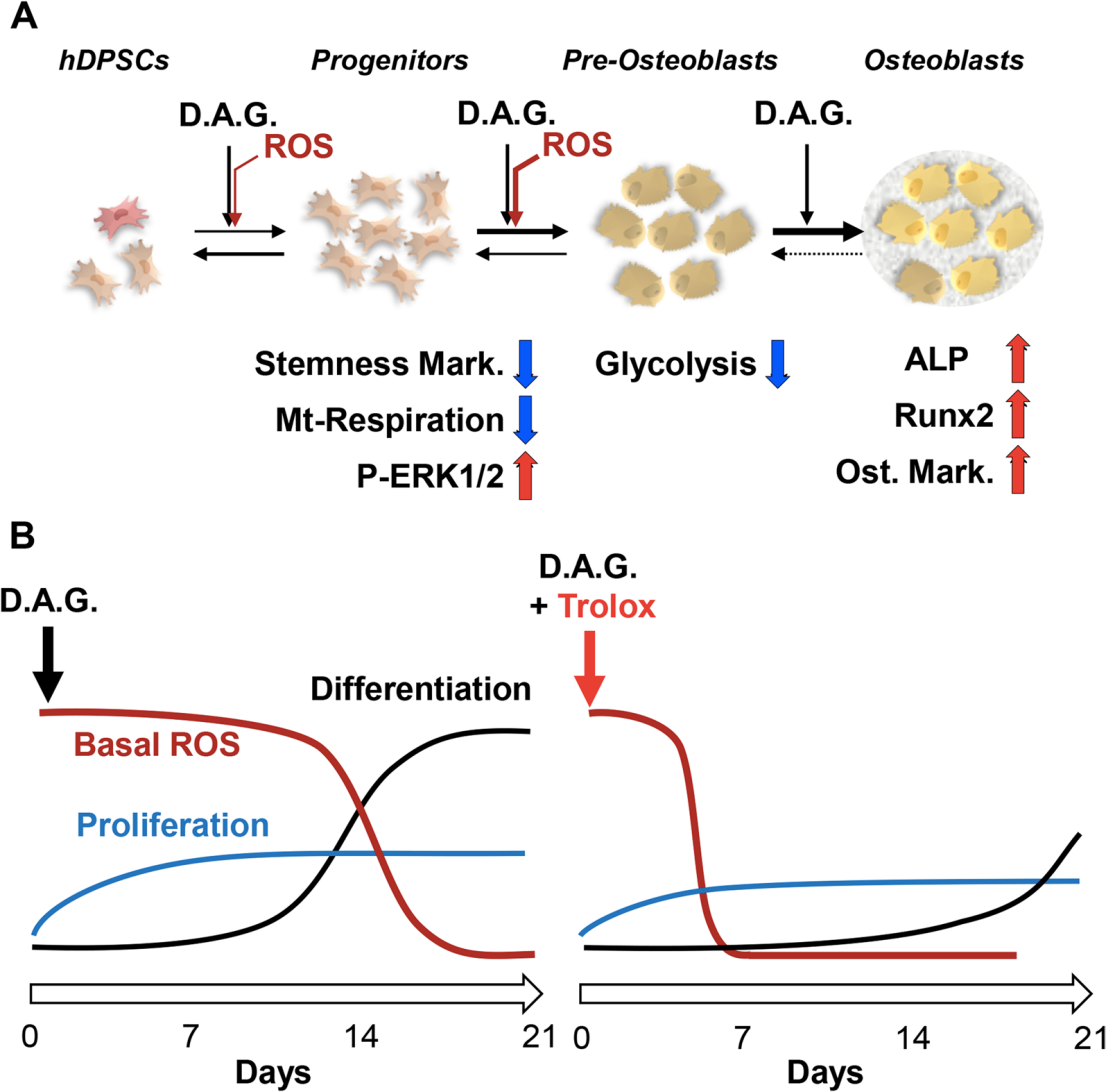
Proposed model of events occurring during osteogenic differentiation of hDPSCs. The scheme shown describes the more relevant observations attained in the present study (**A**) and the effects of Trolox on the interplay between basal ROS level, proliferation and differentiation of hDPSCs (**B**). See Discussion for explanation of the proposed model. hDPSCs: Dental Pulp Mesenchymal Stem Cells; D.A.G.: Dexamethasone, Ascorbate, β-glycerophosphate; ROS: Reactive Oxygen Species; ALP: alkaline phosphatase; Ost.Mark.: Osteoblastic Markers

The scheme shown in Fig. 9 recapitulates these notions along with the main findings attained in the present study and highlights the possible role of redox signaling in this context.

## Conclusions

It is proposed that the physiological level of ROS (likely peroxide species) generated in hDPSCs co-operates with the signaling, triggered by the osteogenic inducers, accelerating the actuation of the differentiative program. The first- and second-week post-induction appear to be essential. Indeed, the last phase of the process, leading to acquisition of the osteoblast phenotype with mineral deposition, is characterized by the decrease of the ROS levels consistent with the time-regulated over-expression of antioxidants [52]. Counteracting the ROS-mediated signaling by administration of an exogenous antioxidant, such as Trolox, in the earlier-middle phase post-induction delays the action of the osteogenic inducers. Accordingly controversial evidence has been documented about the safety of antioxidant therapies including vitamin E [67, 68] and the recently reported negative correlation between serum tocopherols and bone mineralization [69]. Our findings might have practical consequences/applications in regenerative medicine and stem cell-based therapies and confirm the need of caution in the nowadays extensive utilization of antioxidants as pharmacologic adjuvant in the clinical practice as well as food-supplementation in the everyday life.

### Supplementary Information

**Additional file 1**. Additional Fig S1: Full-length blots of Fig. 1D for the protein expression levels of Runx2; Additional Fig S2: Full-length blots of Fig. 2B for the protein expression levels of OxPhos complexes; Additional Fig S5: Full-length blots of Fig. 5C for the protein expression levels of Runx2; Additional Fig S6: Full-length blots of Fig. 6E for the protein expression levels of OxPhos complexes; Additional Fig S7: Full-length blots of Fig. 7A for the protein expression levels of ERK1/2 during osteogenic differentiation of hDPSCs; Additional Fig S8: Full-length blots of Fig. 8D for the protein expression levels of ERK1/2 in untreated and Trolox-treated cells during osteogenic differentiation

## Data Availability

All data generated or analysed during this study are included in this published article.

## Abbreviations

hDPSCs: Human dental pulp stem cells
OB: Osteoblastic
MSC: Mesenchymal stem cells
ERK: Extracellular signal-regulated kinase
MAPK: Mitogen-activated protein kinase
Ocn: Osteocalcin
ALP: Alkaline phosphatase
Runx2: Runt-related transcription factor 2
ROS: Reactive oxygen species
Trolox: 6-Hydroxy-2,5,7,8-tetramethylchroman-2-carboxylic acid
RANKL: Receptor activator of NF-Kb ligand
FCCP: Carbonyl cyanide 4-(trifluoromethoxy)phenylhydrazone
mtΔΨ: Mitochondrial membrane potential
TMRE: Tetramethylrhodamine, ethyl ester perchlorate
DCF-DA: 2,7-Dichlorofluorescein diacetate
Dspp: Dentin sialo phosphoprotein
OxPhos: Oxidative phosphorylation
2-DG: 2-Deoxy-glucose
NOX: NADPH oxidase
q-RT-PCR: Quantitative reverse transcriptase polymerase chain reaction
MEM: Minimum essential medium
GAPDH: Glyceraldehyde-3-phosphate dehydrogenase
SDS: Sodium dodecyl sulfate
OCR: Oxygen consumption rate
ECAR: Extracellular acidification rate
PBS: Phosphate-buffered saline
FBS: Fetal bovine serum
SEM: Standard error of the mean
