# Supplementary material for: Bioenergetic profile and redox tone modulate in vitro osteogenesis of human dental pulp stem cells: new perspectives for bone regeneration and repair
Source: Stem Cell Res Ther. 2023 Aug 22;14:215. doi: 10.1186/s13287-023-03447-9 (PMC10463344; doi:10.1186/s13287-023-03447-9)

SUPPLEMENTARY INFORMATION

Bioenergetic Profile and Redox Tone Modulate *in vitro* Osteogenesis of Human Dental Pulp Stem Cells: New Perspectives for Bone Regeneration and Repair.

Francesca Agriesti [1, 2] *, Francesca Landini [1], Mirko Tamma [1], Consiglia Pacelli [1], Carmela Mazzoccoli [2], Giovanni Calice [2], Vitalba Ruggieri [2, 3], Giuseppe Capitanio [4], Giorgio Mori [1], Claudia Piccoli [1 * †] and Nazzareno Capitanio [1 * †]

[1] Department of Clinical and Experimental Medicine, University of Foggia, 71122 Foggia, Italy; frances-ca.agriesti@unifg.it (F.A.); francesca.landini@unifg.it (F.L.); mirko.tamma@unifg.it (M.T.); consiglia.pacelli@unifg.it (C.P.); giorgio.mori@unifg.it (G.M.); claudia.piccoli@unifg.it (Claudia Piccoli); nazzareno.capitanio@unifg.it (N.C.);

[2] Laboratory of Pre-Clinical and Translational Research, IRCCS-CROB, Referral Cancer Center of Basili-cata, 85028 Rionero in Vulture, Italy; giovanni.calice@crob.it (G.C.); carmela.mazzoccoli@crob.it (C.M.);

[3] Clinical Pathology Unit, "Madonna delle Grazie" Hospital, Matera, Italy; vitalba.ruggieri@asmbasilicata.it (V.R.).

[4] Department of Translational Biomedicine and Neuroscience “DiBraiN”, University of Bari "Aldo Moro", 70124, Bari, Italy; giuseppe.capitanio@uniba.it.

*** Correspondence**: francesca.agriesti@unifg.it (F.A.); claudia.piccoli@unifg.it (Claudia Piccoli); nazzareno.capitanio@unifg.it (N.C.).

† Claudia Piccoli and Nazzareno Capitanio contributed equally to this work.

Additional Fig. S1. Full-length blots of Figure 1 illustrating protein expression levels of Runx2. The cropped blots shown in **Fig 1.D** of manuscript are indicated by a dashed box in the corresponding full length blot. The arrows indicate protein signals at the expected molecular weight. The full length blot on the left shows the β-actin signal obtained on the same blot of Runx2 signal, which has been stripped and subsequently incubated with B actin antibody.

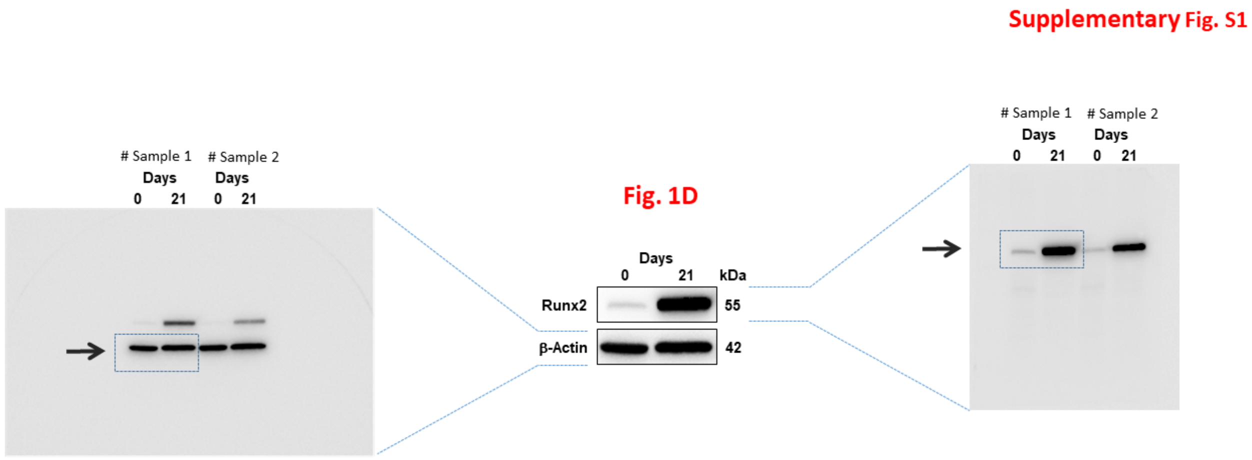

Additional Fig. S2. Full-length blots of Figure 2 illustrating protein expression levels of OxPhos complexes. The cropped blots shown in Fig **2.B** of manuscript belong to the same blot acquired with different exposure times in order to achieve the best signal for each complexes of the MitoProfile antibody cocktail used. Cropped images are indicated by a dashed box in the corresponding full length blot. The arrows indicate protein signals at the expected molecular weight.

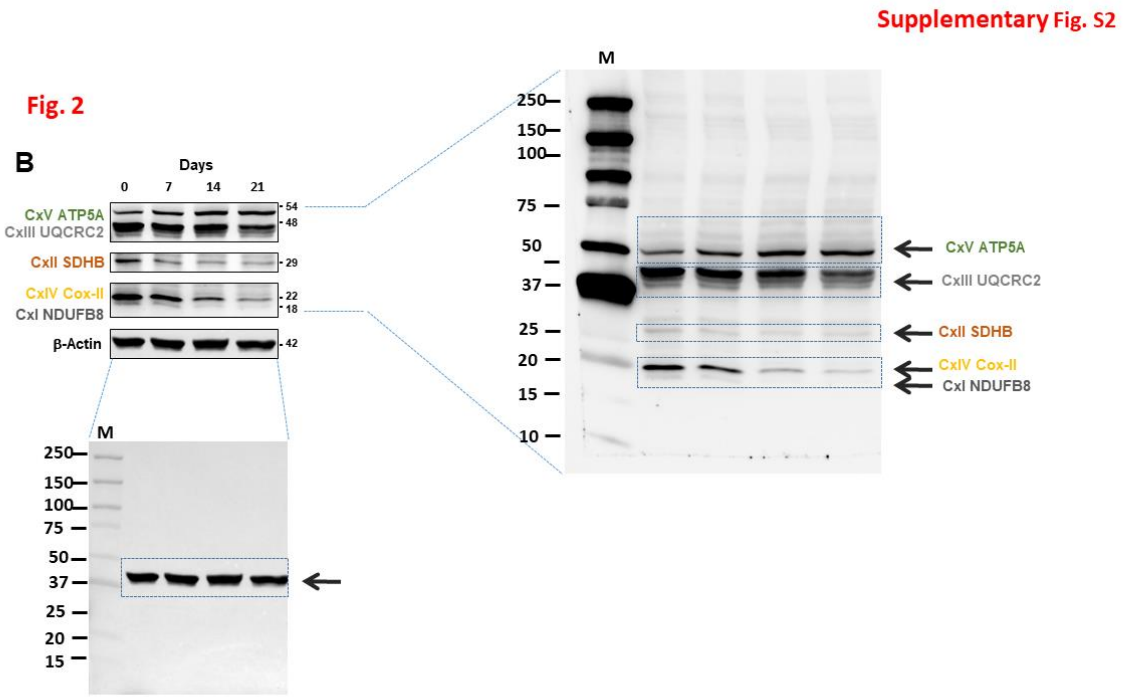

Additional Fig. S5. Full-length blots of Figure 5 illustrating protein expression levels of Runx2 following Trolox treatment. The cropped blots shown in **Fig 5.C** of manuscript are indicated by a dashed box in the corresponding full length blot. The arrows indicate protein signals at the expected molecular weight. M: pre-stained Markers

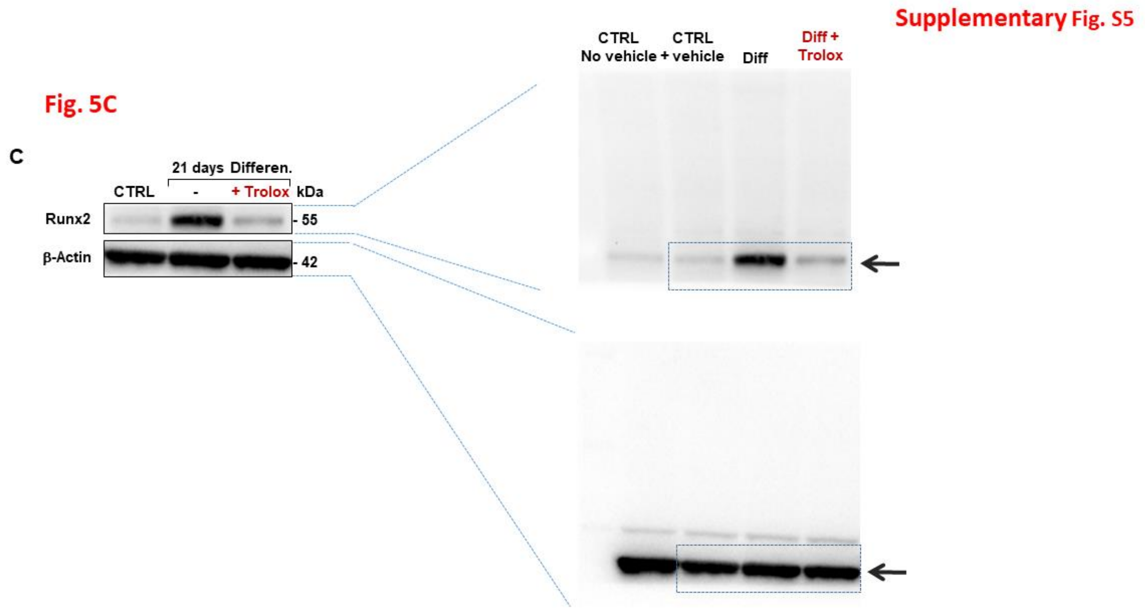

Additional Fig. S6. Full-length blots of Figure 6 illustrating protein expression levels of Oxphos complexes following Trolox treatment. The cropped blots shown in **Fig 6.E** of manuscript are indicated by a dashed box in the corresponding full length blot. The arrows indicate protein signals at the expected molecular weight. M: pre-stained Markers

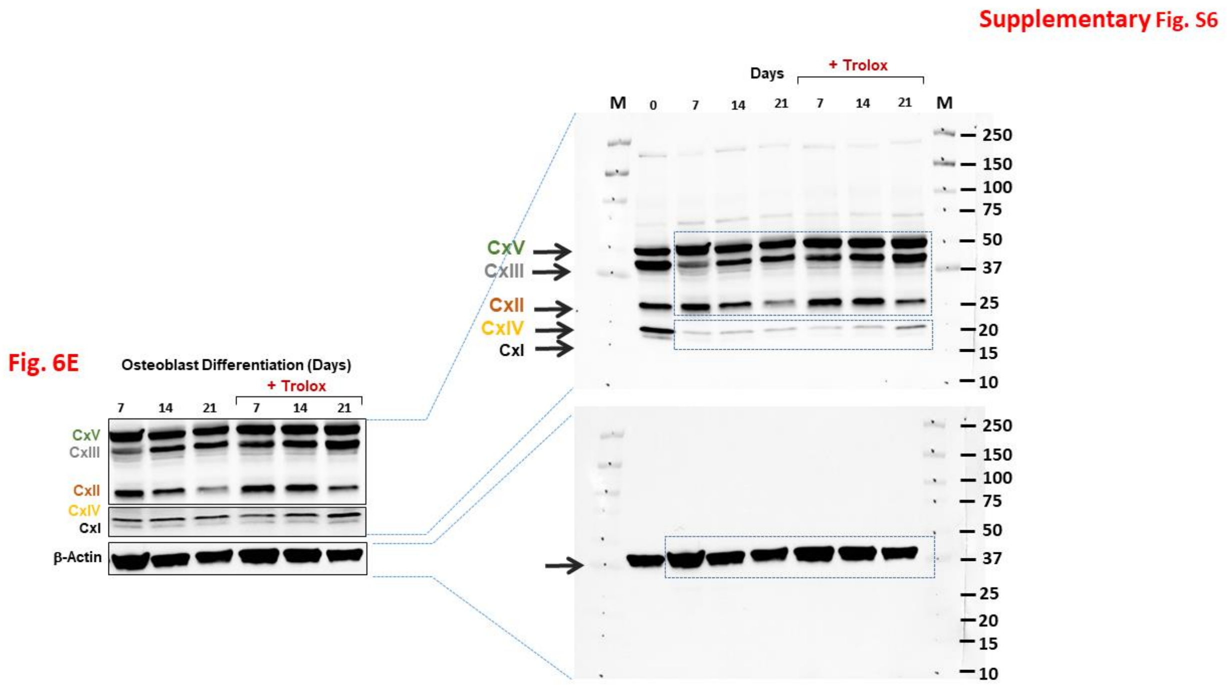

Additional Fig. S7. Full-length blots of Figure 7 illustrating protein expression levels of ERK1/2. The cropped blots shown in **Fig 7.A** of manuscript are indicated by a dashed box in the corresponding full length blot. The arrows indicate protein signals at the expected molecular weight. M: pre-stained Markers

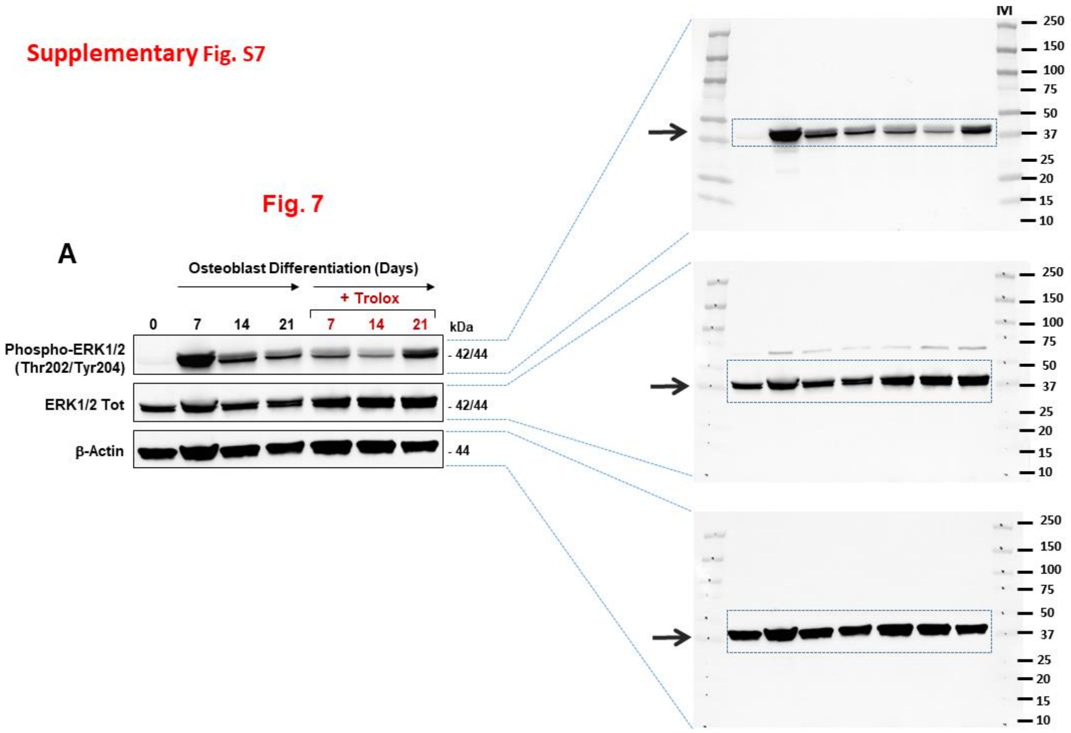

Additional Fig. S8. Full-length blots of Figure 8 illustrating protein expression levels of ERK1/2 following delayed Trolox treatment. The cropped blots shown in **Fig 8.D** of manuscript are indicated by a dashed box in the corresponding full length blot. The arrows indicate protein signals at the expected molecular weight. M: pre-stained Markers.

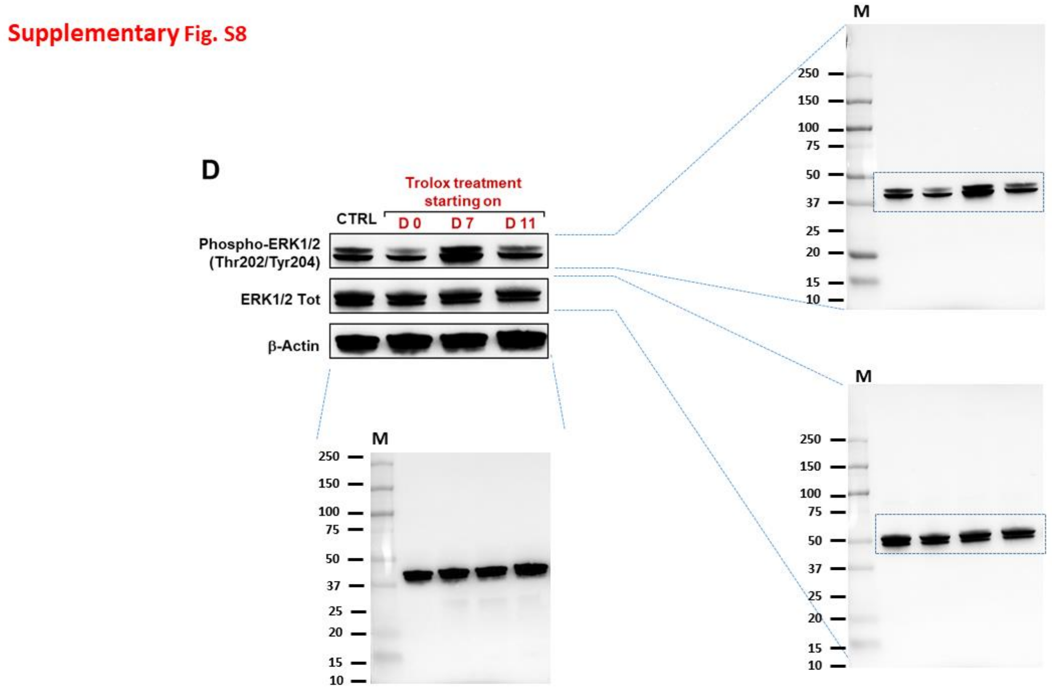

Supplement: Supplementary file 1 — Additional file 1. Additional Fig S1: Full-length blots of Fig. 1D for the protein expression levels of Runx2; Additional Fig S2: Full-length blots of Fig. 2B for the protein expression levels of OxPhos complexes; Additional Fig S5: Full-length blots of Fig. 5C for the protein expression levels of Runx2; Additional Fig S6: Full-length blots of Fig. 6E for the protein expression levels of OxPhos complexes; Additional Fig S7: Full-length blots of Fig. 7A for the protein expression levels of ERK1/2 during osteogenic differentiation of hDPSCs; Additional Fig S8: Full-length blots of Fig. 8D for the protein expression levels of ERK1/2 in untreated and Trolox-treated cells during osteogenic differentiation [file 13287_2023_3447_MOESM1_ESM.pdf]
